# Therapeutic plasma exchange accelerates immune cell recovery in severe COVID-19

**DOI:** 10.3389/fimmu.2024.1492672

**Published:** 2025-01-17

**Authors:** Aurelie Guironnet-Paquet, Hind Hamzeh-Cognasse, Frederic Berard, Fabrice Cognasse, Jean Christophe Richard, Hodane Yonis, Mehdi Mezidi, Olivier Desebbe, Bertrand Delannoy, Sophie Demeret, Clemence Marois, Samir Saheb, Quoc Viet Le, Mathieu Schoeffler, Paul Simon Pugliesi, Sophie Debord, Paul Bastard, Aurélie Cobat, Jean Laurent Casanova, Rémi Pescarmona, Sébastien Viel, Jean François Nicolas, Audrey Nosbaum, Marc Vocanson, Olivier Hequet

**Affiliations:** ^1^ Apheresis Unit, Etablissement Français du Sang Auvergne-Rhône-Alpes, Centre Hospitalier Lyon Sud, Hospices Civils de Lyon (HCL), Pierre Bénite, France; ^2^ International Center for Infectiology Research (CIRI), Université de Lyon, Institut National de la Santé et de la Recherche Médicale (INSERM), U1111, Lyon, France; ^3^ University of Jean Monnet, Mines Saint-Étienne, Institut National de la Santé et de la Recherche Médicale (INSERM), U 1059 SAINBIOSE, Saint-Étienne, France; ^4^ Clinical Immunology and Allergology, Centre Hospitalier Lyon Sud, Hospices Civils de Lyon (HCL), Pierre-Bénite, France; ^5^ Scientific Department, Etablissement Français du Sang Auvergne-Rhône-Alpes, Saint-Etienne, France; ^6^ Intensive Care Unit, Centre Hospitalier Croix–Rousse, Hospices Civils de Lyon (HCL), Lyon, France; ^7^ Department of Anesthesiology and Perioperative Medicine, Sauvegarde Clinic, Ramsay Santé, Lyon, France; ^8^ Neuro-Intensive Care Unit, Assistance Publique des Hopitaux de Paris (AP-HP), Hôpital de la Pitié-Salpêtrière, Paris, France; ^9^ Sorbonne Université, Institut du Cerveau, Paris Brain Institute, Institut du Cerveau et de la Moelle (ICM), Institut National de la Santé et de la Recherche Médicale (INSERM), Centre National de la Recherche Scientifique (CNRS), Assistance Publique des Hopitaux de Paris (AP-HP), Hôpital de la Pitié-Salpêtrière, Departement Médico-Universitaire (DMU) Neurosciences 6, Paris, France; ^10^ Groupe de Recherche Clinique en REanimation et Soins Intensifs du Patient en Insuffisance Respiratoire aiguE (GRC-RESPIRE), Sorbonne Université, Paris, France; ^11^ Hemobiotherapy Unit, Assistance Publique des Hopitaux de Paris (AP-HP), Hôpital de la Pitié-Salpêtrière, Paris, France; ^12^ Intensive Care Unit, Medipôle Lyon Villeurbanne, Villeurbanne, France; ^13^ Department of Anesthesiology and Intensive Care Unit, Centre Hospitalier de Montélimar, Montélimar, France; ^14^ Intensive Care Unit, Centre Hospitalier William Morey, Chalon sur Saône, France; ^15^ Department of Anesthesiology and Intensive Care Medicine, Edouard Herriot Hospital, Hospices Civils de Lyon (HCL), Lyon, France; ^16^ Laboratory of Human Genetics of Infectious Diseases, Necker Branch, Institut National de la Santé et de la Recherche Médicale (INSERM) U1163, Necker Hospital for Sick Children, Paris, France; ^17^ Paris Cité University, Imagine Institute, Paris, France; ^18^ St. Giles Laboratory of Human Genetics of Infectious Diseases, Rockefeller Branch, The Rockefeller University, New York, NY, United States; ^19^ Pediatric Hematology-Immunology and Rheumatology Unit, Necker Hospital for Sick Children, Assistance Publique des Hopitaux de Paris (AP-HP), Paris, France; ^20^ Howards Hugues Medical Institute, New York, NY, United States; ^21^ Immun Monitorage Laboratory, Centre Hospitalier Lyon Sud, Hospices Civils de Lyon (HCL), Pierre-Bénite, France; ^22^ Plateforme de Biothérapies et de production de Médicaments de Thérapie Innovante (MTI), Hôpital Edouard Herriot, Hospices Civils de Lyon (HCL), Lyon, France

**Keywords:** COVID-19, therapeutic plasma exchange, immune response, anti-type I IFN autoantibodies, cytokine storm, adaptive immunity

## Abstract

**Background:**

Immunological disturbances (anti-type I IFN auto-antibody production, cytokine storm, lymphopenia, T-cell hyperactivation and exhaustion) are responsible for disease exacerbation during severe COVID-19 infections.

**Methods:**

In this study, we set up a prospective, randomised clinical trial (ClinicalTrials.gov ID: NCT04751643) and performed therapeutic plasma exchange (TPE) in severe COVID-19 patients in order to decrease excess cytokines and auto-antibodies and to assess whether adding TPE to the standard treatment (ST, including corticosteroids plus high-flow rate oxygen) could help restore immune parameters and limit the progression of acute respiratory distress syndrome (ARDS).

**Results:**

As expected, performing TPE decreased the amount of anti-type I IFN auto-antibodies and improved the elimination or limited the production of certain inflammatory mediators (IL-18, IL-7, CCL2, CCL3, etc.) circulating in the blood of COVID-19 patients, compared to ST controls. Interestingly, while TPE did not influence changes in ARDS parameters throughout the protocol, it proved more effective than ST in reversing lymphopenia, preventing T-cell hyperactivation and reducing T-cell exhaustion, notably in a fraction of TPE patients who had an early favourable respiratory outcome. TPE also restored appropriate numbers of CD4+ and CD8+ T–cell memory populations and increased the number of circulating virus-specific T cells in these patients.

**Conclusion:**

Our results therefore indicate that the addition of TPE sessions to the standard treatment accelerates immune cell recovery and contributes to the development of appropriate antiviral T-cell responses in some patients with severe COVID-19 disease.

## Introduction

Coronavirus disease 2019 (COVID-19), caused by the acute respiratory syndrome coronavirus 2 (SARS-CoV-2), has resulted in widespread global morbidity and mortality over the past 5 years (1). The reasons why some patients developed severe COVID-19 infection and were hospitalised in intensive care units (ICU) whereas other developed minor symptoms, are still not fully understood. The key findings of numerous exploratory studies suggest that an inability to mount a timely antiviral immune response and control the SARS-CoV-2-driven inflammatory response was at the root of the severe viral pneumonia, hypoxemic respiratory failure, coagulopathy and multiorgan damage recorded in severe patients (2–6).

In 20% of cases, especially in the elderly, the progression to critical illness involved a defect in type I IFN due to the presence of anti-IFN auto-antibodies (auto-Abs) (in 15-20% of cases), while other cases in younger adults could be explained by genetic defects (1-5% of cases), which failed to control viral replication and led to a dramatic accumulation of inflammatory monocyte-macrophages and neutrophils at infected sites (3, 4, 7, 8). The latter then secreted large amounts of pro-inflammatory cytokines/chemokines (IL-6, IL-8, TNF-α, IL-1β CXCL10) resulting in major vascular leakage and a state of hyperinflammation, also known as the cytokine storm (7, 9).

In addition, while early, polyfunctional SARS-CoV-2-specific T-cell and antibody (Ab) responses were associated with more rapid viral clearance, less severe disease and a good prognosis, delayed, uncoordinated and maladaptive responses were reported in hospitalised patients (2, 10, 11). Perhaps the most striking dysfunction reported was the profound lymphopenia seen in 80% of patients with severe COVID-19, affecting all T-cell subsets as well as NK cells (12–15). T-cell lymphopenia in these patients may have been driven by T-cell apoptosis as a result of the hyperinflammation state. Numerous inflammatory cytokines such IL-6 and TNF-α are widely known to induce apoptosis in T lymphocytes when administered at high doses *in vitro*, particularly in activated lymphocytes (16, 17). In several longitudinal studies, blood levels of T lymphocytes were shown to be the best correlate of clinical outcome (18–22). Lymphopenia and its severity were associated with a poor prognosis and were therefore considered a reliable predictor of the course of severe COVID-19 infection (19–21). Alternatively, significant variations in T-cell differentiation patterns were also described in hospitalised patients, including a drastic decrease in naive T cells and parallel increases in terminally effector-differentiated (Temra) CD8+ T cells as well as sustained and prolonged lymphocyte activation and proliferation, with higher percentages of exhausted (PD1+Tim3+) or exhausted/senescent (PD1+CD57+) CD4+ and CD8+ T cells (11, 13, 23, 24). Finally, deficiencies in type 1 immune responses were reported in these patients illustrating that the dysfunctional T-cell response failed to control the virus and precipitated disease severity, as suggested by studies in preclinical models using SARS-CoV-2 (25).

A wide range of anti-inflammatory therapies including corticosteroids, JAK inhibitors or biologics such as anti-IL-6 or anti-TNF-α were then used in ICU patients to limit systemic inflammation and multi-organ damage caused by persistent infection and inflammation (26, 27). An original approach known as therapeutic plasma exchange (TPE) also consisted in repeatedly purging the patient’s plasma with plasma from healthy donors (28–38). Performed when patients arrived in ICU, TPE sessions were aimed at removing excess inflammatory mediators as well as pathogenic auto-Abs to attenuate the hyperinflammation state reported and subsequent disturbances in innate and adaptive immunity (29–33, 36–38). This approach also has the advantage of avoiding adaptive response inhibition compared to therapies such as corticosteroids or JAK inhibitors. Hence, TPE showed modest but significant efficacy in different case reports, series, controlled trials and few randomised trials during severe COVID infections, resulting in improvements in oxygenation parameters, multi-organ failure score and mortality rates as well as improvements in inflammation parameters (28–38). However, few studies have reported to date on the impact of TPE on the main immunological disturbances recorded in severe COVID-19 patients (29, 31–33).

In this study, we then conducted a prospective randomised clinical trial and performed TPE in COVID-19 patients arriving in ICU after the onset of acute respiratory distress syndrome (ARDS). We assessed whether performing TPE sessions could help restore the key immune parameters associated with an effective T-cell response and limit progression of the acute respiratory distress syndrome (ARDS).

## Patients and methods

### Clinical study design

Twenty-one patients with severe COVID-19 admitted to 7 different French ICUs (Centre Hospitalier Croix–Rousse, Lyon; Hôpital Edouard Herriot, Lyon; Clinique de la Sauvegarde, Lyon; Medipôle, Lyon Villeurbanne; Centre Hospitalier, Montélimar; Centre Hospitalier William Morey, Chalon-sur-Saône; Hôpital de la Pitié-Salpêtrière, Paris) at the onset of acute respiratory distress syndrome (ARDS) were enrolled in this study from April 2021 to October 2022, namely during the first six waves in Europe.

All of these patients displayed moderate ARDS as well as significant inflammatory syndrome. The primary outcome of the study was the absence of need for intubation at day 10. The secondary clinical outcomes were the oxygenation parameters during 10 days and oxygen supply as well as survival at 2 months while the secondary biological outcomes were the improvements in inflammatory parameters, in cytokine involved in cytokine storm and anti-type I IFN auto-Abs (at day 4) or cellular adaptive immune parameters (at day 7). Inclusion criteria included age over 18 years, Covid-19 infection proven by polymerase chain reaction (PCR) or pulmonary scanner, ARDS with a PaO2/Fi02 index between 75 and 175 mmHg, steroid treatment (at least 2 x 6 mg dexamethasone) and a biological inflammatory state defined by a plasma concentration of at least 2 inflammatory biomarkers above normal values including C-reactive-protein (CRP) > 100 mg/L, Procalcitonin (PCT) > 2 ug/L, Fibrinogen > 8 g/L, D-dimers > 3000 ng/mL and Ferritin > 1000 ng/mL. Exclusion criteria included intubation, incurable cancer, a severe infectious disease such as HIV, body mass index > 40, IgA deficiency, anti-IgA Abs, inability to obtain appropriate central venous access, haemodynamic instability and pregnancy.

After randomisation, patients received 2 different treatment protocols: (i) Ten patients (TPE 1 to TPE 10) received 3 additional early TPE sessions in addition to usual ICU care (including corticosteroids and high-flow oxygen) from day 1 to day 3, (ii) the remaining 11 patients (ST 11 to ST 21) received usual care only named here standard treatment (ST) (**Supplementary Figure S1**). Patient demographics and individual clinical features are presented in **Supplementary Table S1**. Mean age, percentages of predisposing factors and initial oxygenation, inflammatory or prothrombotic parameters were shown in the different cohort groups (**Table 1**). It should be noted that the clinical and biological results were characterised considering the unfavourable or favourable early outcome of the corresponding patient (**Supplementary Figure S2A**). A favourable early outcome was defined for patients who had a low cumulative FiO2 index (< 280 AUC, corresponding to an average low oxygen supply of 40% per day) received between days 4 and 10, as well as no intubation at day 10, whereas an unfavourable early outcome was defined using the opposite criteria.

**Table 1:** Patient’s demographics, clinical features and treatments at baseline – Statistics.

	TPE arm	ST arm	p
**Gender (M/F)**	9/1	9/1	ns
**Age**			
mean +/- SD	66+/-13	62+/-11	ns
median	67	64	
min - max	38-87	43-76	
Comorbidities (number of patients)			
Overweight	4	5	
HTA	4	3	
Diabetes	4	1	
Vascular disease	1	0	
Dyslipidemia	0	1	
Pulmonary disease	1	0	
Autoimmunity	0	1	
**Oxygenation parameters**			
**PaO2/FiO2 (%)**			
mean +/- SD	113+/-28	118+/-32	ns
median	104	124	
min - max	83-165	57-169	
**FiO2 (%)**			
mean +/- SD	63+/-13	64+/-10	ns
median	60	70	
min - max	50-93	50-80	
**Inflammatory parameters**			
**CRP (mg/L)**			
mean +/- SD	160+/-104	65+/-28	p<0.01
median	118	67	
min - max	62-398	8-101	
**PCT (µg/L)**			
mean +/- SD	0.42+/-0.64	0.20+/-0.27	ns
median	0.22	0.11	
min - max	0.00-2.20	0.04-1.00	
**Ferritin (µg/L)**			
mean +/- SD	1246+/-978	1596+/-1023	ns
median	1513	1629	
min - max	13-2880	396-3753	
**Procoagulant parameters**			
**Fibrinogen (g/L)**			
mean +/- SD	6.74+/-1.77	6.73+/-0.96	ns
median	6.55	6.89	
min - max	4.10-10.00	4.63-7.90	
**D-Dimers (ng/ml)**			
mean +/- SD	2545+/-2517	701+/-421	p<0.01
median	1309	550	
min - max	574-7650	354-1640	
**Associated treatment (number of patients)**			
Dexamethasone	10	11	
Tocilizumab	2	2	
	(TPE7,TPE10)	(ST18,ST20)	
Vaccination antiCOVID	1	2	
	(TPE 6)	(ST15, ST19)	

CRP, C-Reactive Protein.

PCT, Procalcitonin.

ns, non significant.

This clinical study (ClinicalTrials.gov ID: NCT04751643) has been approved by several authorities including a French national ethical committee (2020/138) and the Agence Nationale de Sécurité du Médicament et des Produits de Santé (French National Agency for the Safety of Medicines and Health Products) (ANSM; HPSAEC1-202-12-00005). Written informed consent was obtained from each participant or family. Additional information was also retrieved from the electronic medical records after informing the patient or their family, lead ICU physicians and the local ethics committee.

### Therapeutic plasma exchange sessions

The TPE method used was developed by TerumoBCT (Lakewood, Co, USA). The Spectra Optia device is EC-labelled for the TPE software and for the sterile disposable. The method exchanged 1.2 plasma volumes of each patient and the plasma removed was replaced by thawed fresh-frozen plasma obtained from healthy donors by the Etablissement Français du Sang.

### Blood sample collection and processing

Blood (25 mL) was collected from each patient at baseline (day 0) and on days 4 and 7 in order to investigate the impact of TPE on inflammation/immune parameters (**Supplementary Figure S1**). The plasma was prepared by centrifugation whereas peripheral blood mononuclear cells (PBMCs) were isolated from whole-blood samples using density gradient centrifugation with lymphocyte separation medium (Eurobio Scientific). Plasma samples were cryopreserved at -20°C and PBMCs at -80°C in accordance with standard procedures.

### Titration of inflammatory mediators in plasma

Various inflammatory cytokines/chemokines circulating in the plasma of patients treated with TPE and STs were titrated at baseline and on day 4: IFN-α, IFN-γ, IL-1RA, IL-6, IL-7, IL-10, IL-18, TNF-α, CCL-2 (MCP1) and CCL-3 (MIP-1α). Plasma concentrations were determined with Simple Plex technology and an ELLA instrument (ProteinSimple), with the exception of IFN-α. Plasma IFN-α concentrations which were determined with a single-molecule array (Simoa) on an HD-1 Analyser (Quanterix) with a commercial kit for IFN-α2 quantification (Quanterix).

C-reactive protein (CRP) (Immunoturbidimetry Roche Cobas^R^pro), PCT (Immunoflurometry, Kryptor Thermo) and ferritin levels (Immunochemiluminescence LOCI Vista Siemens) were assessed by biochemistry laboratories of each of the seven hospitals at baseline and on day 4. Fibrinogen and D-dimers levels (ACL TOP) were assessed at the same time points by the haemostasis laboratories each of the seven hospitals.

### Clinical, oxygenation, lymphocyte count data

Clinical parameters included (i) daily fraction of inspirated oxygen (FiO2) (%) (ii) PaO2/FiO2 oxygenation index determined by biochemistry laboratories at baseline (day 0) and on day 4, (iii) need for intubation between day 0 and day 10, (iv) duration with oxygen supply (between baseline and day 60), and (v) eventual death (between baseline and day 60). (vi) Lymphocyte and neutrophil counts measured at baseline and day 7 were also obtained from haematological laboratories (Sysmex XR-9000 machine).

### Detection of type I IFN auto-Abs

The neutralizing activities of anti–IFN-α2, anti-IFN-β and anti–IFN-ω auto-Abs were determined in TPE- and ST-treated patients at baseline and on day 4 using a reporter luciferase activity assay, as previously described (8). HEK293T cells were transfected with a plasmid containing the *Firefly* luciferase gene under the control of the human *ISRE* promoter in the pGL4.45 backbone, and a plasmid constitutively expressing *Renilla* luciferase for normalisation (pRL-SV40). Cells were transfected in the presence of the X-tremeGene9 transfection reagent (Sigma-Aldrich) for 24 hours. Cells in Dulbecco’s modified Eagle medium (DMEM, Thermo Fisher Scientific) supplemented with 2% foetal calf serum and 10% healthy control or patient serum/plasma (after inactivation at 56°C for 20 minutes) were either left unstimulated or were stimulated with IFN-α2 (Miltenyi Biotec), IFN-ω (Merck), at 10 ng/mL or 100 pg/mL, or IFN-β (Miltenyi Biotec) at 10 ng/mL, for 16 hours at 37°C. Each sample was tested once for each cytokine and dose. Finally, cells were lysed for 20 minutes at room temperature and luciferase levels were measured with the Dual-Luciferase^®^ Reporter 1000 assay system (Promega) according to the manufacturer’s protocol. Luminescence intensity was measured with a VICTOR-X Multilabel Plate Reader (PerkinElmer Life Sciences). *Firefly* luciferase activity values were normalised against *Renilla* luciferase activity values. These values were then normalised against the median induction level for non-neutralising samples and expressed as a percentage. Samples were considered neutralising if luciferase induction, normalised against *Renilla* luciferase activity, was below 15% of the median values for controls tested on the same day.

### Mass cytometry analysis

After thawing, PBMCs collected from TPE- and ST-treated patients at baseline and on day 7 as well as from 10 healthy donors (Etablissement Français du Sang Auvergne-Rhône-Alpes) were consecutively stained for viability discrimination with Cisplatin (Standard BioTools). Fc-receptors were blocked (Fc Blocking, Miltenyi Biotec) and barcoded to discriminate and identify each sample of the corresponding patients. Cadmium-labelled CD45 Abs (Standard BioTools) were employed as the barcode using a 6-choose-3 format in order to enable sample multiplexing. After extracellular barcoding, the stained samples were combined as a single multiplexed sample. Thereafter, the sample was stained with extracellular markers. Thirty-six mass cytometry Abs were obtained as preconjugated metal-tagged antibodies from Standard BioTools or generated in-house by conjugating unlabelled, purified Abs (Miltenyi Biotec, Biolegends) to isotope-loaded polymers. A detailed list of the Abs used is provided in **Supplementary Table S2**. The cells were permeabilised using Cytofix/Cytoperm solution (Cytofix/Cytoperm™, BD Biosciences) and then stained with Abs for intracellular staining (Granulysin, Ki-67, FoxP3, Granzyme B) and DNA-stained by an iridium (Ir) intercalator (Standard BioTools). Just before acquisition, cells were diluted tenfold in Four Element Calibration Beads (Standard BioTools). Acquisition was performed and data were recorded using mass cytometry (Fluidigm Helios, CytoF2). Flow Cytometry Standard 3.0 files were imported into FlowJo software v10^®^.

### High-dimensional mass cytometry data analysis

The multiplex sample was debarcoded using single-cell debarcoder software (single-cell debarcoder, Github.com). Cytometry data files were normalised using the bead-bead Fluidigm normalisation algorithm. Files were then manually gated in FlowJo for cells with no beads (Ce140^-^), cleanup (double positive Ir191^+^/Ir193^+^ for DNA) and singlets (Ir191-). CD4+ or CD8+ T-cell data were exported for unsupervised analysis utilising the OMIQ platform (www.omiq.ai). Data were Arcsinh-transformed with a coefficient of 5, which was used within the OMIQ platform. For lineage population analysis, total individual cells were subsampled to 1000 events except for some samples (TPE1, TPE3, TPE6, TPE7 and ST14 on day 0 and TPE3 and ST14 on day 7) for which we used the maximum number of cells available in live CD4+ and CD8+ T cells. Cell data were then run for unsupervised t-distributed stochastic neighbour embedding (t-SNE) and FlowSOM algorithms based on the Euclidean distance and Ward-linkage leading to 10 distinct clusters considering the relative MFI of 25 markers simultaneously: CCR7, CD45RA, CD45RO, CD28, CD127, CD25, FoxP3, CD57, PD1, HLA-DR, CD38, CD69, CD71, CD95, CD39, CD73, TIM3, LAG3, CD122, Ki-67, Granzyme B, Granulysin, KLRG1, NKG2C and NKG2A.

Downstream analyses included standard gating to remove beads, aggregates or dead cells, and further identified main leukocyte subsets after excluding CD14+ and CD19+ cell lineages (**Supplementary Figure S3**). FlowSOM clusters were visualised as heatmaps showing the integrated MFI of each marker per cluster and the individual abundance of each cluster per patient was generated with the OMIQ platform.

### Detection of SARS-CoV-2 specific T cells

To identify SARS–CoV-2 reactive T cells, 1 × 10^6^ PBMCs from TPE- and ST-treated patients collected at baseline and on day 7 were cultured in RPMI 1640 media (ThermoFisher Scientific) containing 0.3 mg/mL glutamine, 100 U/mL penicillin, 0.1 mg/mL streptomycin and 5% human AB serum and in the presence of a mix of membrane glycoprotein (M) and nucleocapsid phosphoprotein (N) peptides or spike glycoprotein (S) peptide only (1 µg/mL) (Miltenyi Biotec) and anti-CD28 (ThermoFisher Scientific) plus anti-CD49d (Biolegend) Abs (both 2 µg/mL) for 6 hours. M, N and S peptides were selected for their capacity to activate both CD4+ and CD8+ restrained T cells of the most frequent HLA-types. Positive control consisted in PMA-ionomycin stimulation (0.01 µg/mL and 1 µg/mL respectively, Sigma). In all stimulations, Brefeldin A (7 μg/mL, Millipore Sigma) and Monensin (1 µg/mL, BD BioSciences) were added after 1 hour, allowing intracellular molecule detection by flow cytometry.

At the end of a 6-hour culture, cells were initially treated with Fc-block and then with live-dead, fluorescently labelled Abs-recognising human CD3, CD4, CD8 and CD154 proteins. They were then permeabilised using Fix/Perm buffer (BD Biosciences, Le Pont de Claix, France) and stained with fluorescent anti-TNF-α and anti-IL-2 Abs (**Supplementary Table S2**). Cells were analysed on an LSR FORTESSA flow cytometer (BD Biosciences) and data were analysed using FlowJo software (v10^®^; FlowJo, Ashland, Oregon, USA).

After pregating on live (live-dead negative cells) lymphocytes, antigen (Ag)-reactive CD4+ T cells were identified based on co-expression of CD154 and IL-2 or TNF-α, while Ag-reactive CD8+ T cells were identified based solely on IL-2 or TNF-α expression. SARS–CoV-2-specific T-cell responses were determined in peptide–stimulated cultures after subtracting the frequency data obtained from unstimulated controls with a minimum of 10 events and 2-fold higher frequencies of CD154+ CD4+TNF-α+ and CD154+CD4+IL-2+ or CD8+TNF-α+ and CD8+IL-2+ T cells compared to the unstimulated control.

### Statistics

Wilcoxon statistical tests (two-tailed tests) were used to compare distributions in quantitative and temporal measures in TPE- and ST-treated groups. A type-1 error rate correction was applied to account for multiple testing. Sidak’s correction was used to control the 5% family error rate and the respective threshold was documented in each/corresponding figure legend.

For exploratory analysis, two separate principal component analyses (PCA) were performed on leukocyte cytokine variation (between day 4 and day 0) and lymphocyte T-cell variation (between day 7 and day 0), respectively. The contribution of each covariate to the first three components was presented. Pearson correlation coefficients between the retained principal components of leukocyte cytokine variation PCA and lymphocyte T-cell variation PCA were calculated.

## Results

### A limited number of early TPE sessions did not change the course of acute respiratory distress syndrome in severely ill COVID-19 patients

Twenty–one patients were consented to the study. Patient demographics and clinical characteristics at baseline are described in **Table 1**. There were no differences in age, gender and oxygenation parameters between the patients enrolled in the TPE and ST groups. However, patients in the TPE group had higher increases in inflammatory (CRP) and prothrombotic (D-dimers) biomarkers than those in the ST group.

We initially investigated whether the addition of 3 early sessions of TPE to conventional treatments improved the course of acute respiratory distress syndrome (ARDS) in severely ill COVID-19 patients. To this end, we examined the need for intubation, the level of oxygenation required (% of FiO2 required, the PaO2/FiO2 index and the total number of days requiring oxygenation) during the first 10 days and 2 months after the start of treatment.

TPE sessions induced only slight side-effects due to the transfusion of thawed fresh-frozen plasma with grade 2 side effects consisting in maculopapular rash regressive after antihistamine treatment, in 5 out of 30 TPE sessions. No thrombo-embolism or infectious complications occurred after the use of the central catheter (used in 8/10 TPE patients).

**Figure 1:**
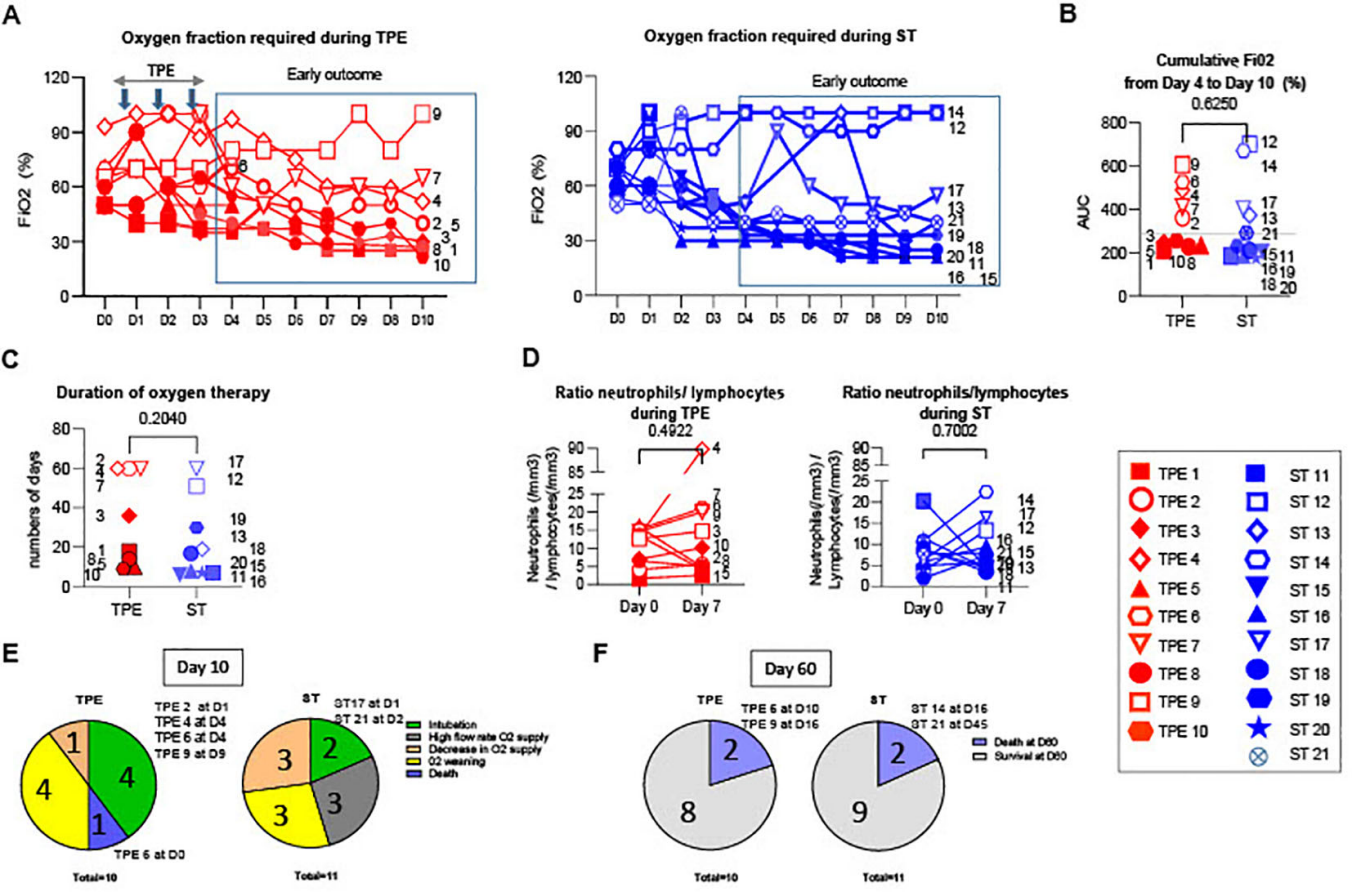
Oxygenation parameters, neutrophils/lymphocytes ratio and numbers of deaths during the study. **(A)** Daily fraction of oxygen (FiO2), **(B)** cumulative FiO2 between day 4 and day 10 and **(C)** duration of oxygen therapy after 2 months in patients treated with TPE (red symbols) or ST (blue symbols). **(D)** Evolution of neutrophils/lymphocytes ratio measured using an automated haematology analyser between baseline and day 7. **(E)** Numbers of deceased, intubated patients and patients with increased or decreased O2 supply and O2 weaning at day 10. **(F)** Numbers of deaths and survival at day 60. In **(A–D)**, patients were further stratified according to unfavourable (empty symbols) and favourable early outcome (full symbols), as defined in Material and Methods and **Supplementary Figure S2**. The number next to each symbol corresponds to the patient's assignment. In **(E, F)**, the days notified in pie charts correspond to the date for each complication. Statistics were calculated with Wilcoxon and an adjusted risk (α’)=0.01.

Six out of eleven patients (ST11, ST15, ST16, ST18, ST19 and ST20) in the ST group and 5/10 patients (TPE1, TPE3, TPE5, TPE8 and TPE10) in the TPE group received a lower oxygen supply in the days after treatment initiation (**Figure 1A**), as illustrated by the low cumulative FiO2 index between day 4 and day 10 (**Figure 1B**) as well as the increased PaO2/FiO2 index at 200 mmHg (**Supplementary Figure S2B**). All of the patients plus ST13 received less than 40 days of oxygenation overall (**Figure 1C**) and were completely weaned off oxygen after 2 months (**Supplementary Table S3**). Interestingly, we observed that these patients had a low blood neutrophile/lymphocyte ratio on day 7, which has been previously associated with favourable/good medium-term prognosis in severe COVID-19 infections (39) (**Figure 1D**).

Conversely, 5 patients in both groups (ST12, ST13, ST14, ST17 and ST21 as well as TP2, TP4, TP6, TP7 and TP9) required a high oxygen flow rate or were intubated before day 10 (**Figures 1A, B, E**; **Supplementary Table S3**). Most of them still required oxygen therapy at day 60 (**Figure 1C**; **Supplementary Table S3**). It should be noted that 2 patients in each group died during the study as the consequence of the ARDS: ST14 and ST21 on day 16 and day 45, and TPE6 and TPE9 on day 10 and day 16, respectively (**Figure 1F**; **Supplementary Table S3**).

These results therefore indicate that the progression of ARDS in patients with severe COVID was highly variable in both groups. Some patients showed a favourable early outcome associated with a progressive decrease in O2 supplementation (< 280 AUC day 4 - day 10 cumulative FiO2 index) during treatment and a low neutrophil/lymphocyte ratio on day 7 (**Figures 1A, D**; **Supplementary Figure S2**), while others were still suffering from ARDS after 2 months. Some even died in the meantime. These results provide further evidence that the addition of a limited number of early TPE sessions had no major impact on the clinical course of ARDS symptoms in this small cohort of COVID-19 patients.

### TPE sessions removed circulating type I IFN-neutralising auto-Abs

Despite the lack of effect on respiratory parameters and survival rates, we nevertheless wondered whether TPE sessions might have modulated the main immunological disturbances that characterise severe COVID-19 infections.

**Figure 2:**
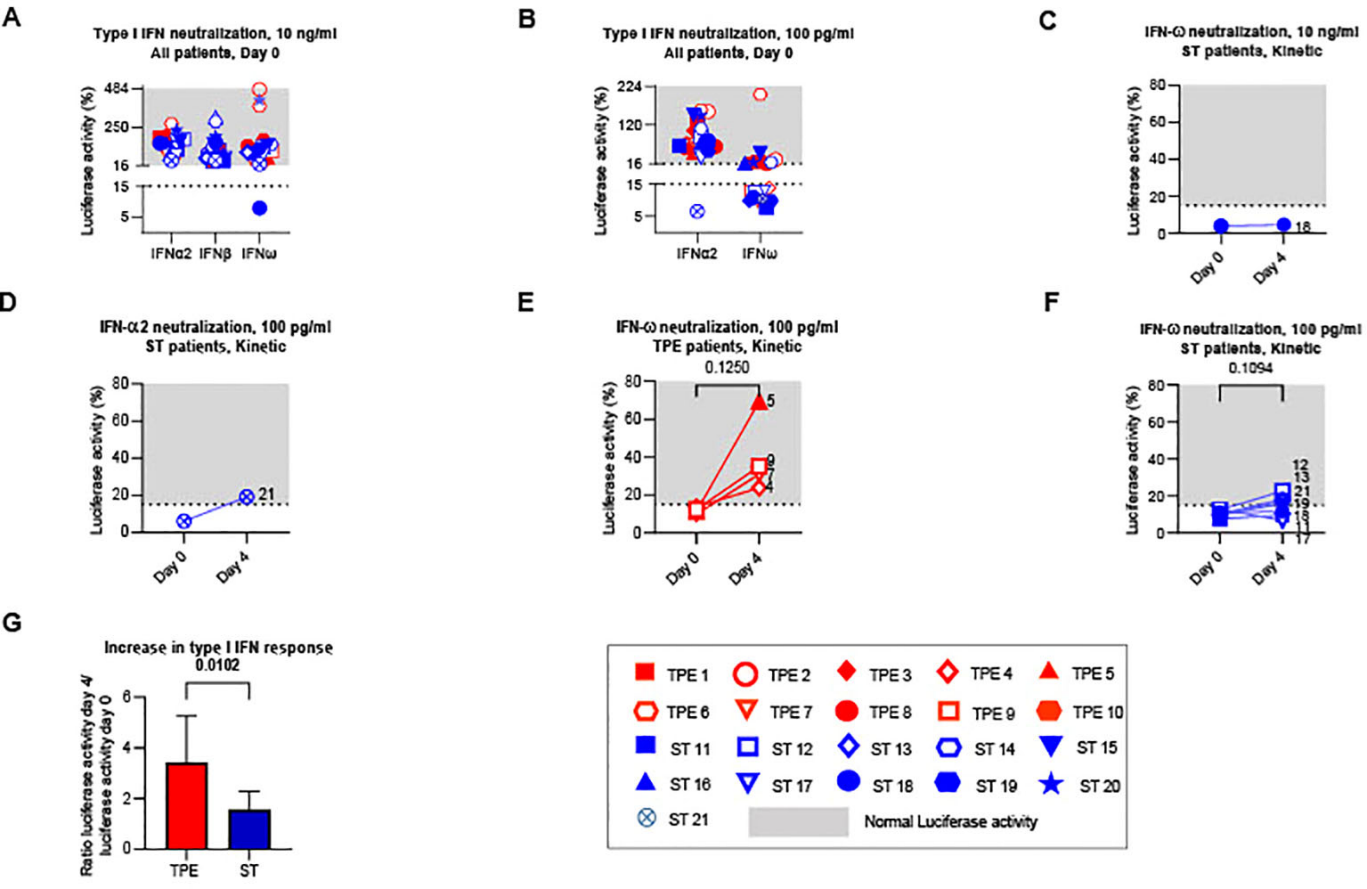
Type I IFN neutralizing auto-Abs circulating in the plasma of TPE- and ST-treated patients between baseline and day 4. The presence of type I IFN neutralizing auto-Abs present in the plasma of TPE (red symbols)- and ST (blue symbols)-treated patients was quantified using a reporter cell-based neutralization assay and HEK293T cells transfected with a luciferase plasmid containing interferon-stimulated response elements. **(A, B)** Luciferase activity in HEK293T cells stimulated with low (A, 10 ng/mL) or high (B, 100 ng/mL) concentrations of IFNα2, IFNβ and IFNω and the plasma from TPE (red symbols)- or ST (blue symbols)-treated patients collected at baseline. **(C–F)** Changes in luciferase activity at day 4 are also shown for patients with significant type I IFN neutralizing auto-Abs (IFNω, **C, E, F**; IFNα2, **D**) detected at baseline. A luciferase activity below or above 15% was used to reflect respectively the presence or the absence (grey area) of anti-type I IFN auto-Abs. **(G)** The evolution of type I IFN neutralizing activity in the two groups of patients between day 4 and baseline was appreciated by dividing the luciferase activity values (IFNα2 100 pg/ml, IFNω 10 ng/ml and 100 pg/ml) on day 4 by those on day 0. In **(A–F)**, patients were further stratified according to unfavourable (empty symbols) and favourable early outcome (full symbols). The number next to each symbol corresponds to the patient's assignment. Statistics were calculated with Wilcoxon and an adjusted risk (α’)=0.017.

We therefore first sought to verify whether TPE sessions modulated the amount of type I IFN-neutralising auto-Abs circulating in the blood of these patients. To this end, we used a reporter cell-based neutralisation assay previously described (8), and HEK293T cells transfected with a luciferase plasmid containing interferon-stimulated response elements and cultured with high or intermediate type I IFN concentrations to measure the presence of various type I IFN auto-Abs (IFNα2, IFNβ, IFNω). In this assay, the quantities of detectable auto-Abs were inversely proportional to luciferase activity. At baseline (on day 0), no anti-IFNβ auto-Abs were detected in any patients, while anti-IFNα2 and anti-IFNω auto-Abs were observed in 1 (ST21) and 11 (ST11, ST12, ST13, ST17, ST18, ST19, ST21, TPE 4, TPE5, TPE 7 and TPE9) patients respectively, as shown by low luciferase activity (<15%) in this functional assay (**Figures 2A, B**). It should be noted that auto-Abs levels were low in almost all patients since neutralisations were reported at substrate concentrations of 100 pg/mL of type I IFN (**Figure 2B**) as opposed to 10 ng/mL (**Figure 2A**), with the exception of patient ST18.

Treatment impact on type I IFN-neutralising auto-Ab concentrations was then analysed the day after the last TPE session. As expected, we observed increased luciferase activity in the 4 patients with anti-IFNω Abs who received TPE compared to controls who received ST (**Figures 2C–G**), indicating that TPE acutely removed circulating type I IFN-neutralising Abs in the early stages of severe COVID-19 infection in these patients.

### TPE sessions reduced or limited key inflammatory mediator concentrations in the plasma of TPE- patients

We then examined whether performing TPE also reduced the levels of various key inflammatory mediators circulating in the blood of these patients.

**Figure 3:**
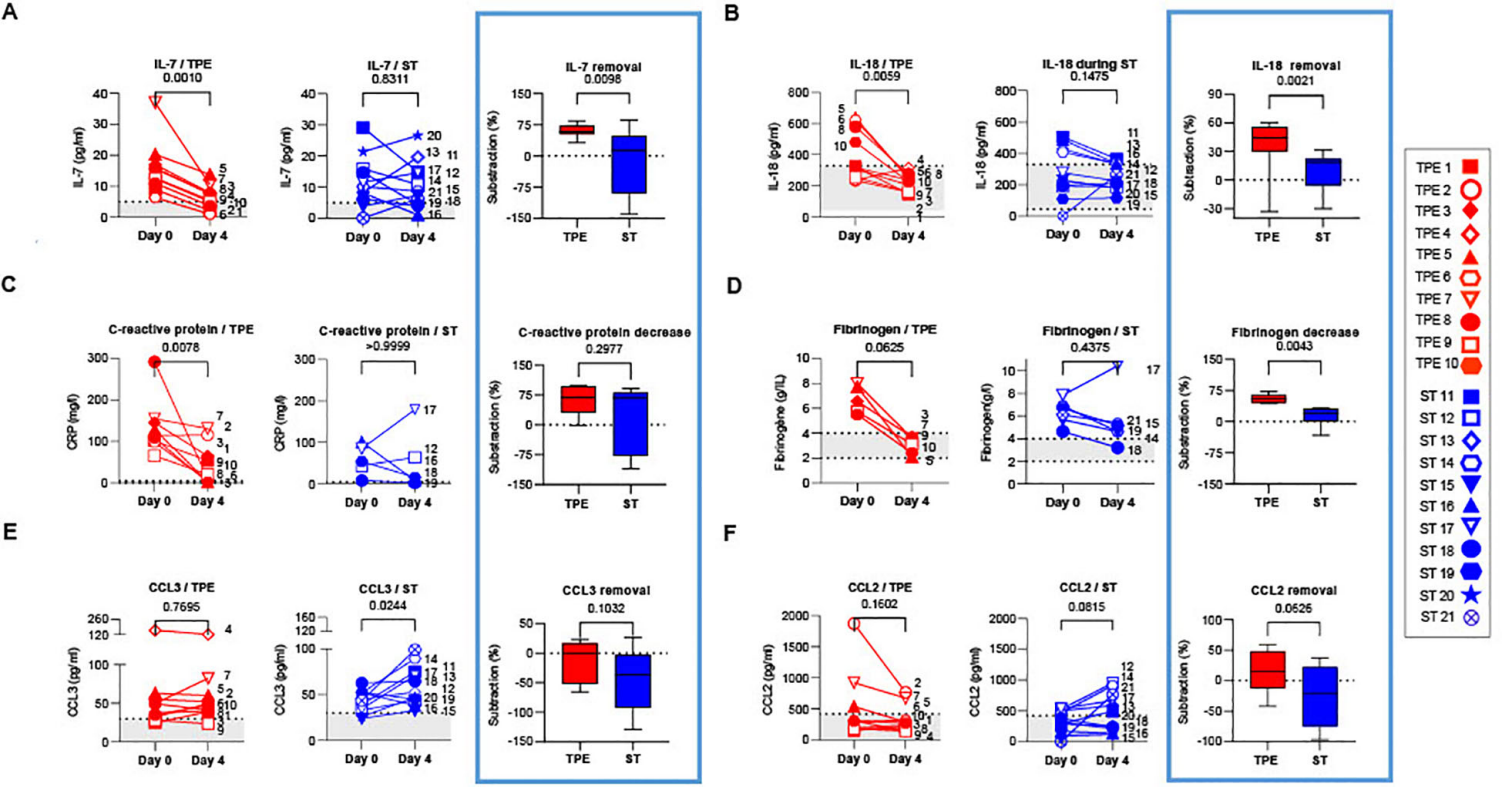
Concentrations of plasma cytokines/chemokines in TPE- and ST-treated patients between baseline and day 4. IL-7 **(A)**, IL-18 **(B)**, C-reactive protein **(C)**, Fibrinogen **(D)**, CCL3 **(E)** and CCL2 **(F)** concentrations measured by Simpleplex technology and single-molecule array the plasma of TPE (red symbols)- and ST (blue symbols)-treated patients at baseline and day 4. For each mediator and treatment group, a percentage of removal or decrease between baseline and day 4 was calculated as follow = ([concentration mediator X] day 0 - [concentration mediator X] day 4) / [concentration mediator X] day 0. In **(A–F)**, patients were further stratified according to unfavourable (empty symbols) and favourable early outcome (full symbols). The number next to each symbol corresponds to the patient's assignment. The grey areas correspond to the concentrations of mediators usually detected in the plasma of healthy volunteers. Statistics were calculated with Wilcoxon and an adjusted risk (α’)=0.005.

Eight cytokines (IFN-α, IFN-γ, IL-1Ra, IL-6, IL-7, IL-10, IL-18 and TNF-α), two chemokines (CCL2 and CCL3) as well as C-reactive protein and fibrinogen were titrated at baseline and on the day after the last TPE session (day 4). Changes in plasma concentrations were compared between the two treatment groups. Indicative of the onset of a significant inflammatory syndrome, the plasma concentrations of all mediators (with the exception of IFN-γ and CCL2) were found to be significantly elevated in the vast majority of patients (in both the ST and TPE groups) at baseline compared to standard measurements in healthy donors (**Figure 3**; **Supplementary Figure S4**).

Interestingly, while a significant decrease was observed in IFN-α concentrations in both groups on day 4 (**Supplementary Figure S4A**), a sharp drop in IL-7, IL-18, C-reactive protein and fibrinogen concentrations (**Figures 3A–D**), as well as a downward trend in IL-10 (**Supplementary Figure S4B**) were recorded only in the TPE group, with values approaching those of healthy donors. Furthermore, we observed an increase in CCL3 concentrations as well as a tendency for elevated CCL2 and TNF-α levels in the ST group on day 4 *versus* baseline, which was not the case in the TPE group (**Figures 3E, F**; **Supplementary Figure S4C**). No changes were detected for IL-6, IFN-γ and IL-1Ra concentrations (**Supplementary Figures S4D–F**).

Taken together, these results suggest that, in addition to the standard treatment, performing TPE improved the elimination or limited the production of certain inflammatory mediators such as IL-18, C-reactive protein, fibrinogen, CCL2 or CCL3 in the early stages of severe COVID-19 infection. TPE also impacted plasma concentrations of cytokines involved in T-cell survival and functions such as TNF-α, IL-7 and IL-10.

### Rapid normalisation of T cell counts in TPE patients with early favourable respiratory outcome

We subsequently wondered whether the TPE sessions might had stimulated the recovery of different metrics of T-cell immunity. As severe lymphopenia is a major feature in severe COVID infections, we initially analysed the effects of TPE sessions on the recovery of T-cell subsets on day 7 post-baseline.

**Figure 4:**
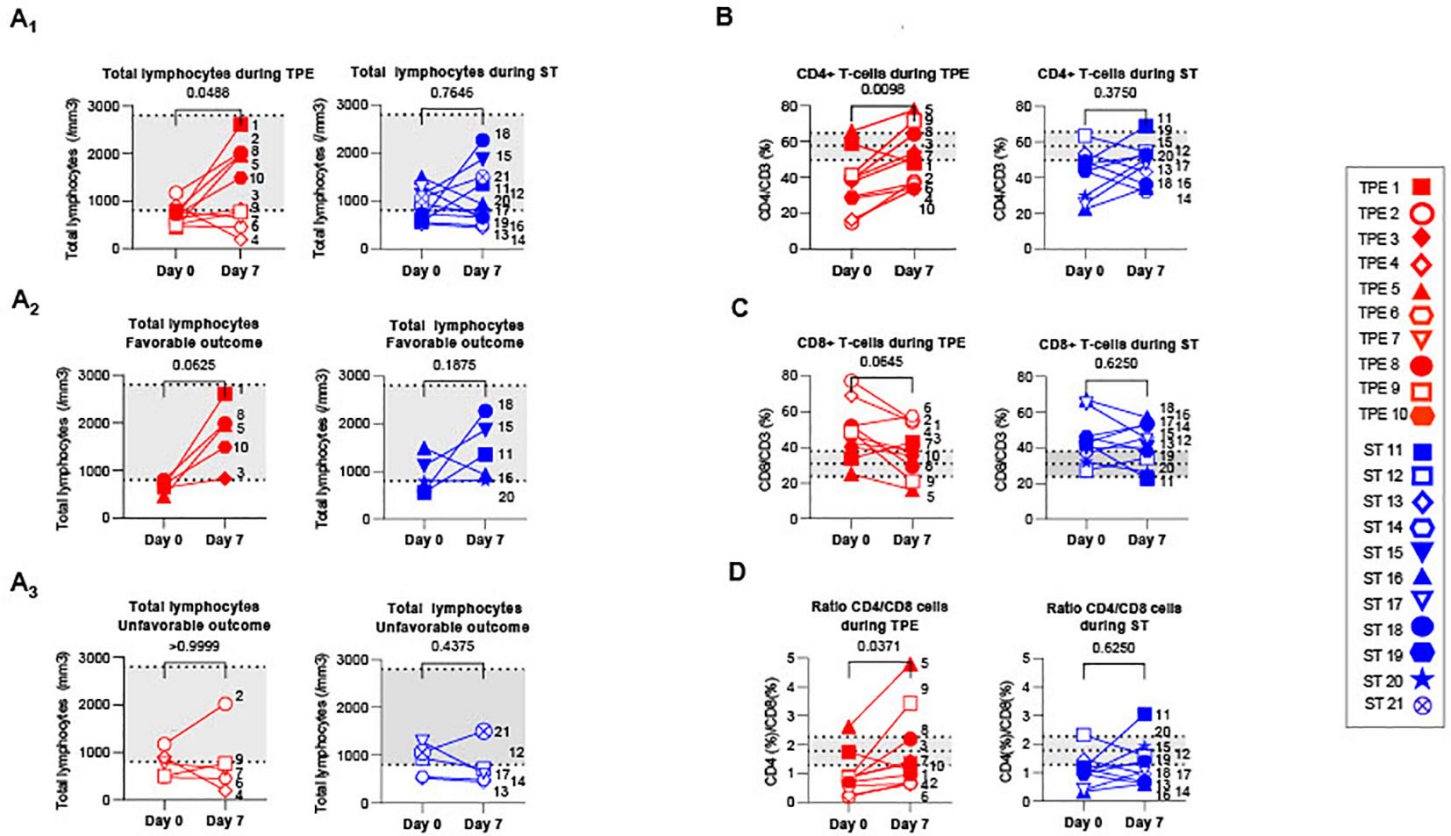
T cell recovery and changes in CD4+ and CD8+ T cell distribution between baseline and day 7. **(A)** Changes in total lymphocyte counts measured using an automated haematology analyser in TPE (red symbols)- and ST (blue symbols)-treated patients between baseline and day 7. Lymphocyte counts data are depicted for all patients (A1) and further detailed for patients with favourable (A2, empty symbols) and unfavourable (A3, full symbols) outcome. **(B–D)** Variations in CD4+ **(B)** and CD8+ **(C)** T-cell frequencies among CD3+ T cell population, as detected by spectral cytometry, and variations in respective CD4+/CD8+ T cell ratio **(D)** are also shown. In **(A–D)**, the number next to each symbol corresponds to the patient's assignment. The grey areas correspond to standard values usually detected in healthy donors **(A)** or mean +/- SD values that we detected in 10 healthy volunteers **(B–D)**. Statistics were calculated with Wilcoxon and an adjusted risk (α’)=0.005.

In this instance, 14 patients in the cohort, including 6 in the ST group and 8 in the TPE group, had lymphocyte counts below a standard threshold (800 lymphocytes/mm^3^ of blood usually detected in heathy donors by routine haematological labs) at baseline (**Figure 4A1**). Besides, as described in numerous viral infections, the distribution of T-cell subsets was skewed towards a predominance of CD8+ T cell subsets over their CD4+ counterparts, as revealed by the decreased frequency of CD4+ (**Figure 4B**) and increased frequency of CD8+ (**Figure 4C**) T fractions, and resulting modulation of the CD4/CD8 ratio (**Figure 4D**) compared to control values recorded in 10 heathy volunteers.

Interestingly, a significant increase in lymphocyte count was found in the TPE group, but not in the ST group, 7 days after initiating the protocol (**Figures 4A2, A3**). Normalisation of lymphocyte counts was observed, in particular, in all patients presenting a favourable respiratory outcome on day 10 (TPE1, TPE3, TPE5, TPE8 and TPE10) (**Figure 4A2**), but not in those still requiring a high oxygen intake at that time (TPE4, TPE6, TPE7, TPE9) (**Figure 4A3**). It should be noted that lymphocyte counts remained low or even decreased in ST patients (ST12, ST13, ST14 and ST17) who presented unfavourable outcome on day 10 (**Figures 4A2, A3**).

A substantial increase in the percentage of CD4+ T cells (**Figure 4B**) and a resulting inversion in the CD4/CD8 T-cell ratio (**Figure 4D**) was also noted on day 7 in virtually all TPE group patients but not in the ST group. Combining the frequency data with the elevated lymphocyte count at this time point highlights a significant rise in the CD4+ T cell count in the TPE-treated group (**Supplementary Figure S5A**).

Collectively, these results demonstrate that TPE sessions helped regulate T-cell homeostasis in the days following the start of treatment. They accelerated the recovery of T-cell counts in patients with an early favourable respiratory outcome.

### TPE sessions substantially modified the nature of the T-cell response

We then explored and compared the phenotypic identity of CD4+ and CD8+ T-cell subsets present in the blood of COVID-19 patients at baseline and on day 7 in a bid to determine how TPE impacted the ongoing T-cell response.

**Figure 5:**
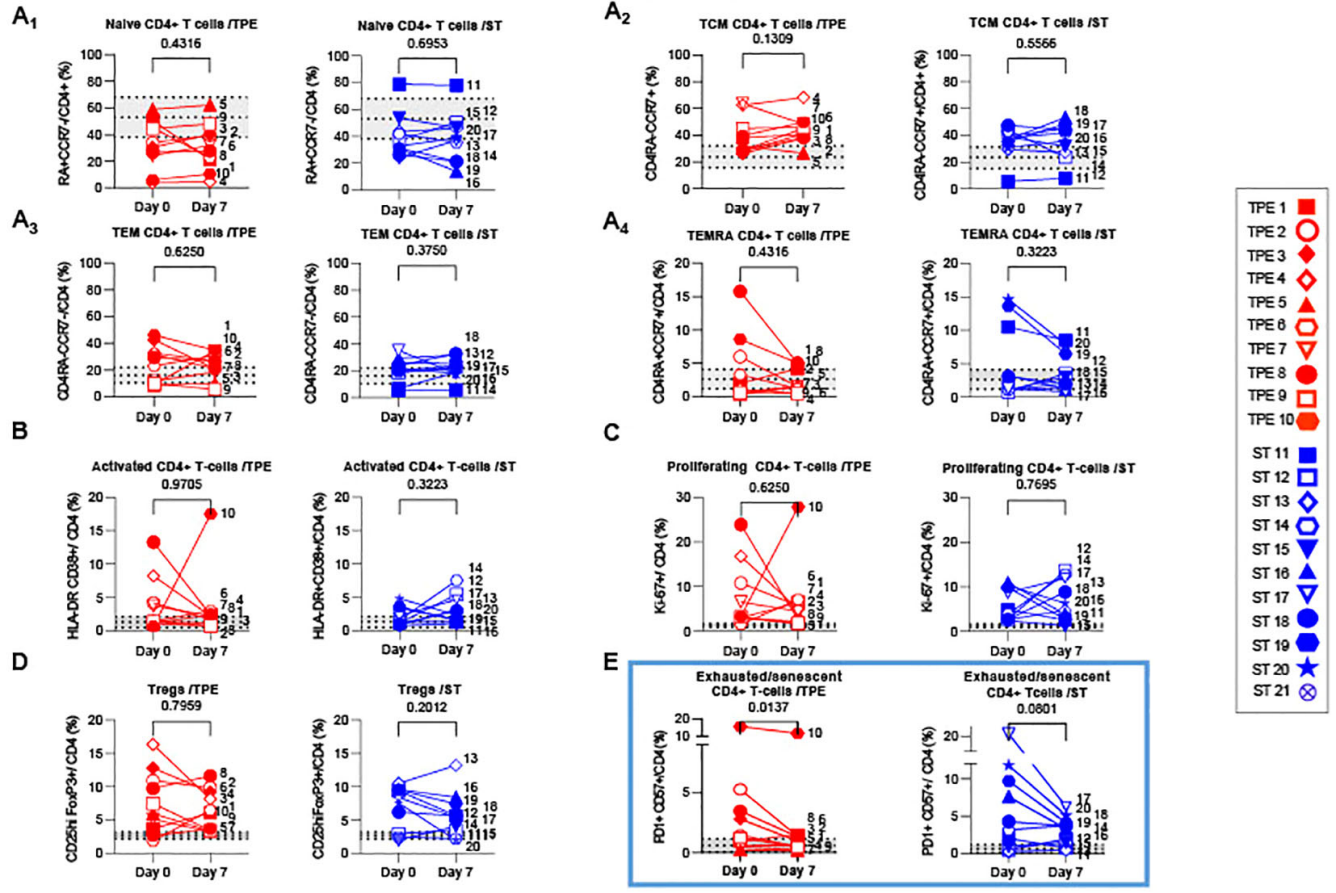
Variations in differentiation, activation, exhausted/senescent and regulatory phenotype in the CD4+ T cell population from baseline to day 7. **(A)** Changes in the frequencies of naïve (A1, CD45RA+CCR7+), central memory (A2, Tcm, CD45RA-CCR7+), effector memory (A3, Tem, CD45RA-CCR7-) and terminally effector memory (A4, Temra, CD45RA+CCR7-) subsets among CD4+ T cell population measured by spectral cytometry in TPE (red symbols)- and ST (blue symbols)-treated patients between baseline and day 7. **(B–E)** Changes in the frequencies of activated (**B**, HLADR+CD38+), proliferating (**C**, Ki67+), regulatory (**D**, FoxP3+CD25+) and exhausted/senescent (**E**, PD1+CD57+) subsets among CD4+ T cell population are also shown. In **(A–E)**, patients were further stratified according to unfavourable (empty symbols) and favourable early outcome (full symbols). The number next to each symbol corresponds to the patient's assignment. The grey areas correspond to mean +/- SD values detected in 10 healthy volunteers. Statistics were calculated with Wilcoxon and an adjusted risk (α’)=0.012.

We initially examined variations in frequencies and numbers of naïve, central memory (Tcm), effector memory (Tem) and effector memory re-expressing CD45RA (Temra) T-cell subsets, by analysing the expression of classical CD45RA and CCR7 markers (**Figures 5A**; **Supplementary Figure S6A**). As expected, we observed a predominance of both CD4+ and CD8+ memory T-cell subsets in the majority of patients at baseline, including a high frequency of CD4+Tcm or CD4+Tem (**Figures 5A2-A3**), and CD8+Tem or CD8+Temra (**Supplementary Figures S6A2-3**), compared to healthy volunteer data. Interestingly, while the distribution of naïve/memory phenotype did not change among the CD4+ T-cell fraction on day 7 (**Figures 5A1–A4**), an increase in CD8+Tem was observed in the ST-treated group at this point as well as a parallel decrease in CD8+Temra frequency in both groups (**Supplementary Figures S6A3-4**). Finally, the respective numbers of naive and memory cells found was essentially influenced by the afore-mentioned lymphocyte counts thus highlighting a predominant increase in naive CD4+ and CD8+ T cells, CD4+ Tcm and Tem on day 7 in the TPE group compared to baseline (**Supplementary Figures S5B1-3**, **S5D1-3**).

We also investigated the presence of activated, proliferating and senescent/exhausted CD4+ and CD8+ T cells, as well as regulatory FoxP3+CD25+CD4+ T cells (Tregs) (**Figures 5B–E**; **Supplementary Figure S6B–D**). Activated and senescent/exhausted cells were detected on the basis of joint expression of HLA-DR and CD38 or PD-1 and CD57 markers respectively, while proliferating cells were identified on Ki-67 protein expression. To avoid confusion between activated and exhausted cells, we analysed CD57+PD1+ T cells as senescent/exhausted T cells, not only PD1+, to reflect the activation status. As expected, variable, albeit high frequencies of activated, proliferating and/or exhausted/senescent CD4+ and CD8+ T cells were observed in a large majority of COVID-19 patients at baseline, when compared against values detected in healthy volunteers (**Figures 5B–E**; **Supplementary Figure S6B-D**). A higher proportion of Tregs was also observed (**Figure 5D**). Most of these phenotypic metrics did not change on day 7, notably the percentage of proliferating CD8+ and CD4+ T cells which remained relatively high (25 +/- 13 and 6 +/- 6%, respectively). In contrast, frequencies of senescent/exhausted CD4+PD-1+CD57+ dropped only after TPE treatment (**Figure 5E**).

**Figure 6:**
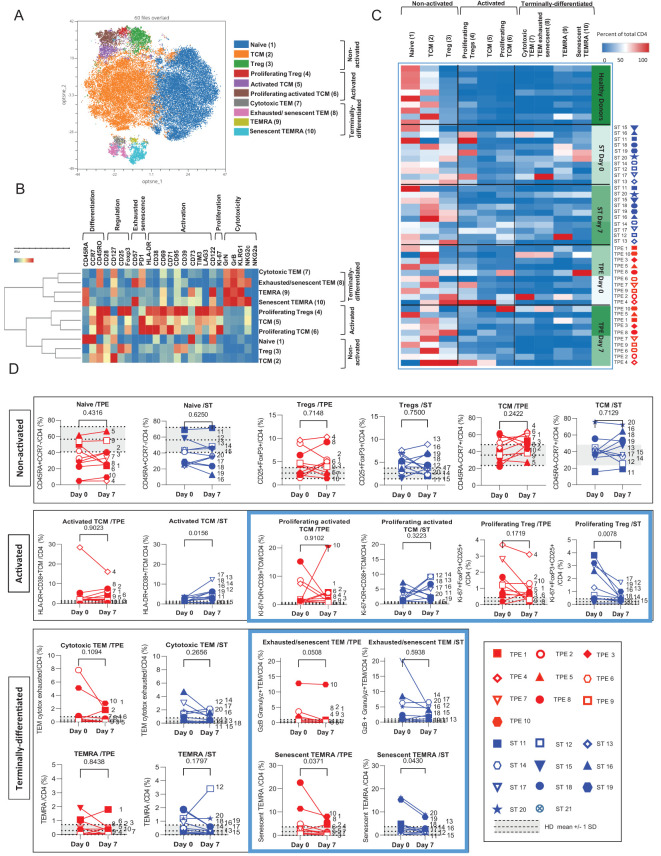
High dimensional cell analysis of CD4+ T cell subsets during the study. FlowSOM analysis with automatic consensus clustering was performed on concatenated CD4+ T cell data (1000 cells/sample) from TPE- and ST-treated patient samples collected at baseline and day 7. Data obtained from 10 healthy volunteers were also included as controls. **(A)** Results were presented as a self-organizing map gathered in 10 background coloured clusters (1-10). Each cluster includes phenotypically similar cells. **(B)** Heat map of the integrated MFI of 25 markers across the 10 FlowSOM clusters identified in **(A)** The colour in the heatmap represents the median of the arcsinh for each cluster (centroid) transformed with a coefficient of 5 for marker expression. Clusters (lines) were hierarchically metaclustered using Ward’s method, and differential marker expression was used to assign each cluster and metacluster with a specific identity. **(C, D)** Cluster frequencies were determined for each sample from each patient and each healthy volunteer and presented as a heatmap, in which the colours represent cluster abundance among the CD4+ T cell population **(C)**. Results are also depicted as individual scatter plots, in which the grey areas correspond to mean +/- SD values detected in the 10 healthy volunteers. TPE (red symbols)- and ST (blue symbols)-treated patients were also stratified according to unfavourable (empty symbols) and favourable early outcome (full symbols). The number next to each symbol corresponds to the patient's assignment. Statistics were calculated with Wilcoxon and an adjusted risk (α’)=0.005.

**Figure 7:**
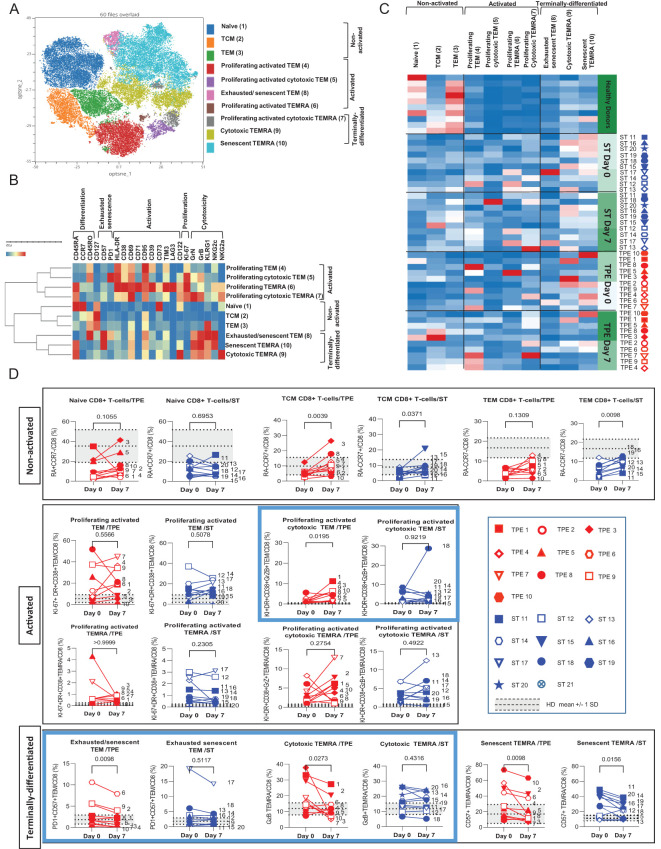
High dimensional cell analysis of CD8+ T cell subsets during the study. FlowSOM analysis with automatic consensus clustering was performed on concatenated CD8+ T cell data (1000 cells/sample) from TPE- and ST-treated patient samples collected at baseline and day 7. Data obtained from 10 healthy volunteers were also included as controls. **(A)** Results were presented as a self-organizing map gathered in 10 background coloured clusters (1-10). Each node includes phenotypically similar cells. **(B)** Heat map of the integrated MFI of 25 markers across the 10 FlowSOM clusters identified in **(A)** The colour in the heatmap represents the median of the arcsinh for each cluster (centroid) transformed with a coefficient of 5 for marker expression. Clusters (lines) were hierarchically metaclustered using Ward’s method to group subpopulations with similar phenotype, and differential marker expression was used to assign each cluster and metacluster with a specific identity. **(C, D)** Cluster frequencies were determined for each sample from each patient and each healthy volunteer and presented as heatmap, in which the colours represent cluster among the CD8+ T cell population **(C)**. Results were also depicted as individual scatter plots, in which the grey areas correspond to mean +/- SD values detected in the 10 healthy volunteers. TPE (red symbols)- and ST (blue symbols)-treated patients were also stratified according to unfavourable (empty symbols) and favourable early outcome (full symbols). The number next to each symbol corresponds to the patient's assignment. Statistics were calculated with Wilcoxon and an adjusted risk (α’)=0.005.

To take our analysis one step further, we then performed high-dimensional profiling and scrutinised the (co)-expression of 25 different markers (CD45RO, CD45RA, CCR7, CD28, CD127, CD25, FoxP3, PD-1, CD57, Tim3, LAG3, NKG2a, NKG2c, HLA-DR, CD38, CD69, CD71, CD122, Ki-67, Granulysin, Granzyme B, KLRG1, CD95, CD39 and CD73) on both CD4+ and CD8+ T-cell populations. Using concatenated spectral cytometry data from all the ST- and TPE-treated samples collected at baseline and on day 7, we ran FlowSOM, a self-organising map (SOM) clustering algorithm, to assess the heterogeneity of CD4+ and CD8+ T-cell populations present in the different patients (**Figures 6A**, **7A**). FlowSOM data were merged into clusters, the identity of which was determined based on the integrated median fluorescence intensity (iMFI) values of each differentiation, activation, proliferation, senescence/exhaustion and regulatory marker (**Figures 6B**, **7B**).

Hence, the CD4+ T-cell population was stratified into 10 clusters including 3 clusters of Tcm cells, 2 clusters of Tem, Temra or of Tregs cells and 1 cluster of naive cells (**Figure 6A**). The 3 CD45RO+CD45RA-CCR7+ Tcm clusters and the 2 FoxP3+CD25+ Tregs clusters were further discriminated based on the expression of an activated (high expression of CD71, CD38, CD69, PD-1, and/or HLA-DR markers) and/or proliferating (Ki67) phenotype (**Figure 6B**). In contrast, the 2 CD45RO+CD45RA-CCR7-Tem clusters and CD45RO+CD45RA+CCR7-Temra clusters were distinguished by the differential expression of exhaustion/senescence CD57 and PD-1 markers as well as NKG2c (**Figure 6B**). Hierarchical clustering stratified the 10 clusters into 3 meta-clusters including non-activated (naive, Tcm, Tregs), activated/proliferating (Tregs and Ki67^high^ or Ki67^low^ Tcm) and terminally-differentiated (PD1+CD57+NKG2C+ or PD1+CD57-NKG2C- Tem and CD57+NKG2C+ or CD57-NKG2C- Temra) T-cell subsets.

By analysing the frequencies of the 10 CD4+ T cell clusters, we observed that the significant increase in both activated, proliferating and/or terminally-differentiated values detected in total CD4+ T cells at baseline versus the control values from healthy volunteers, was the result of increased percentages of Tcm (cluster 5: Ki67^low^Tcm and cluster 6: Ki67^high^Tcm), Tem (cluster 7: PD1+CD57-NKG2C-Tem and cluster 8: PD1+CD57+NKG2C+Tem) and Temra clusters (cluster 9: CD57-NKG2C-Temra and cluster 10: CD57+NKG2C+Temra) (**Figures 6C, D**). While no major changes in subset frequencies were recorded on day 7 for the vast majority of clusters, a significant decrease in proliferating Tregs and an increased frequency of Ki67^low^Tcm were observed in the ST group but not in the TPE group (**Figure 6D**). Finally, FlowSOM analyses confirmed the decrease in CD4+ Tem co-expressing the exhaustion/senescence markers PD1+ and CD57+ on day 7 only in the TPE group, and in senescent CD57+NKG2C+Temra in both groups (**Figure 6D**).

Similar to its CD4+ counterpart, the CD8+ T-cell population was subsequently stratified into 10 different cell clusters and discriminated based on the expression of differentiation markers (CD45RA, CD45RO and CCR7) with high or low levels of activation (HLA-DR, CD38, CD69, CD71 and CD95) and cytotoxicity (Granzyme B, Granulysin) markers before being stratified into 3 hierarchic meta-clusters of non-activated, activated/proliferating and terminally-differentiated T-cell subsets. Non-activated T cells included naive, Tcm and Tem cell subsets (clusters 1, 2 & 3, respectively) (**Figures 7A, B**). Activated/proliferating cell subsets encompassed cytotoxic (Granzyme+ and Granulysin^High/Int^) and non-cytotoxic (Granzyme- and Granulysin-) Tem (clusters 4&5) and Temra (clusters 6&7) cells (**Figures 7A, B**). Finally, terminally-differentiated cell subsets contained exhausted/senescent PD1+CD57+ Tem (Cluster 8), cytotoxic but non-proliferating Temra (Cluster 9) and senescent PD1-CD57+ Temra cells (cluster 10) (**Figure 7B**).

Once again, FlowSOM analyses comparing the frequencies of the 10 cell groups in baseline and day 7 samples from ST- and TPE-treated patients confirmed initial observations performed at CD8+ T-cell population level with variable but dramatic increases in the frequencies of several activated, proliferating and/or senescent/exhausted Tem and Temra clusters (clusters 4-10) at baseline when compared with control values for healthy volunteers (**Figures 7C, D**). The main changes in subset frequencies recorded on day 7 were an increase in the percentage of activated-proliferating and cytotoxic Tem (cluster 5) cells and a decrease in the percentage of exhausted/senescent Tem (cluster 8) or cytotoxic Temra (cluster 9) cells in the TPE- and non-ST-treated groups. An increase in non-activated Tcm (cluster 2) cells and a decrease in senescent Temra (cluster 10) cells were also observed in both groups (**Figures 7C, D**).

Collectively, these results show that, 7 days after the start of treatment, strong activation/proliferation of CD4+Tem, CD8+Tem and CD8+Temra cell subsets were still evident in COVID-19 patients. This was apparent in the 2 treatment groups whereas TPE prevented activation of CD4+ Tcm. However, these findings also indicate that TPE sessions helped to reshape the ongoing immune response, as illustrated by significant changes in the distribution of memory T-cell subsets with a decrease in exhausted/senescent Tem cells compared to ST.

### Increased frequencies of spike-specific T cells in TPE-treated COVID-19 patients

**Figure 8:**
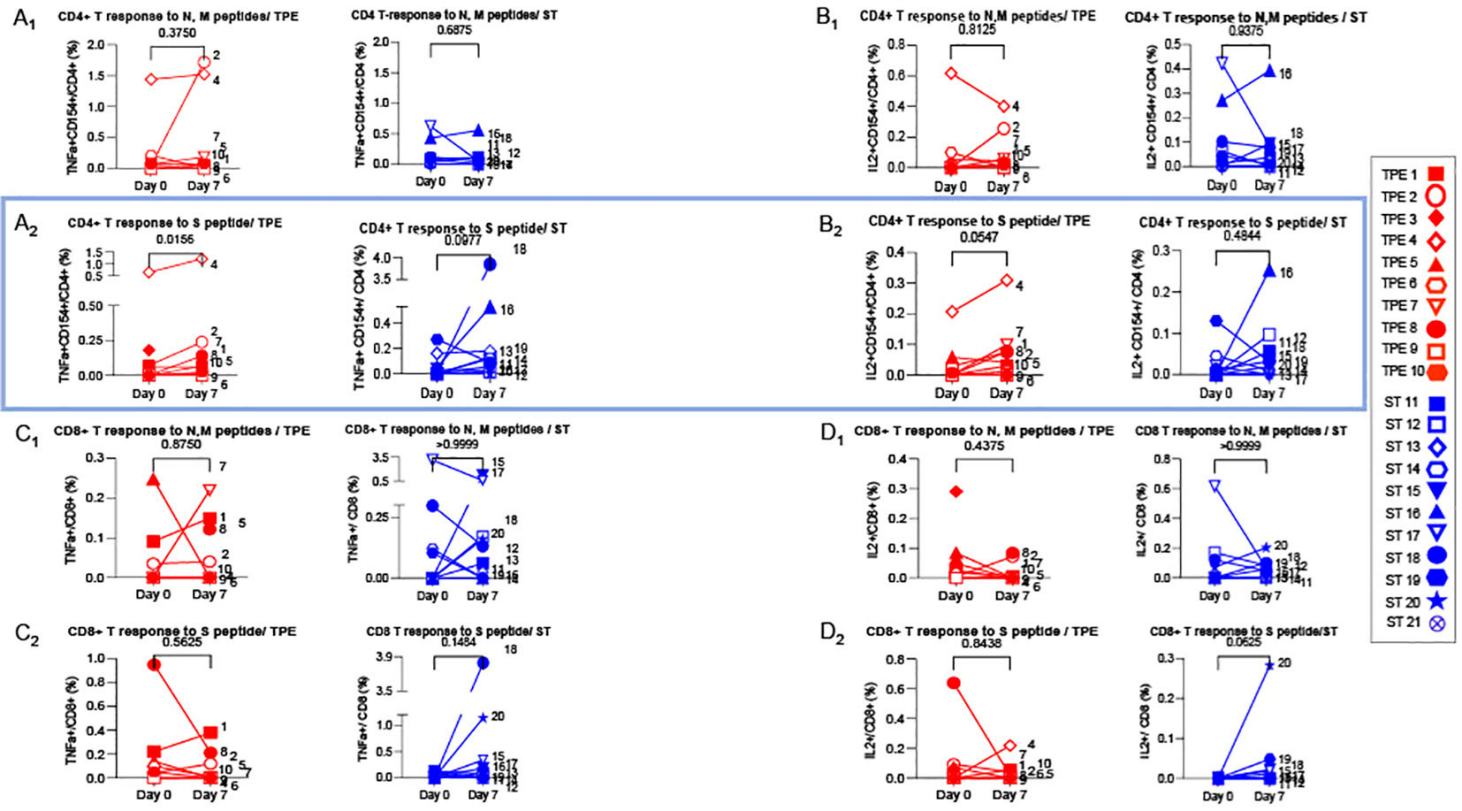
Virus-specific CD4+ and CD8+ T cell-responses detected at baseline and day 7. 1x106 PBMC collected from TPE (red symbols)- and ST (blue symbols)-treated patients at baseline and day 7 after the start of treatments were restimulated *in vitro* for 6 hours with N & M (A1-D1) or S (A2-D2) peptides from SARS-COV2 virus, plus anti-CD28+ and anti-CD49d+ mAbs. The percentages of effector cells expressing TNF-α (A1-A2, C1-C2) or IL-2 (B1-B2, D1-D2) cytokines in both CD4+CD154+ **(A, B)** or total CD8+ **(C, D)** T cells were determined at the end of the stimulation period by flow cytometry. In **(A–D)**, patients were further stratified according to unfavourable (empty symbols) and favourable early outcome (full symbols). The number next to each symbol corresponds to the patient's assignment. Statistics were calculated with Wilcoxon and an adjusted risk (α’)=0.006.

We then wondered whether TPE sessions could also have stimulated the recovery a potent virus-specific T-cell response. To this end, PBMCs collected at baseline and on day 7 after starting treatments were restimulated with peptides specific for spike glycoprotein (S) or membrane glycoprotein (M) and nucleocapsid phosphoprotein (N) from SARS-COV-2 virus for 5 days, and the percentages of effector cells expressing TNF-α or IL-2 cytokine in both CD4+CD154+ or total CD8+ T cells were determined by fluorescence-activated cell sorting (FACS) (**Figures 8A–D**). Low but substantial frequencies of specific CD4+ and CD8+ T cells were detected in the blood of COVID-19 patients compared to healthy donors, both at baseline and on day 7, thus highlighting the onset of an anti-viral response in COVID-19 patients (**Figures 8A–D**). Importantly, significant increases in the frequencies of spike-responding TNF-α+ and IL-2+ CD4+CD154+ T cells were detected in the group of patients treated with TPE on day 7 compared to baseline (**Figures 8A2-B2**).

These results therefore suggest that TPE sessions might have contributed to strengthening the antiviral response in COVID-19 patients.

### T lymphocyte improvements correlated to cytokine/chemokine removal

**Figure 9:**
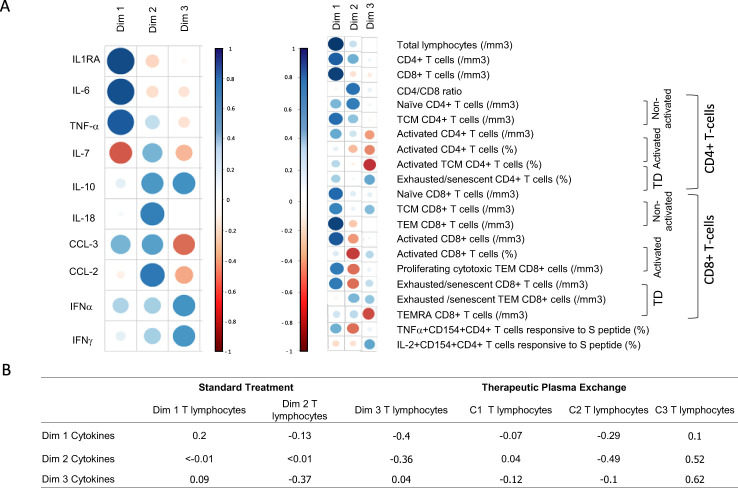
T lymphocyte improvements correlated to cytokine removal **(A)** Comparison of variation in immune parameters on all patients (treated by TPE or ST). Principal component analysis performed on the variation cytokine (values at day 0 – values at day 4) to variation of T cell parameters (values at day 7 – values at day 0). Results depicted groups of cytokines that varied and groups of T cell parameters that varied. The size of the symbols corresponded to the significance of the correlation while the colour corresponded to the sign of correlation (blue for positive correlation and red for negative correlation). Each dimension defined a group of marker. Together the 3 components shown defined approximately 70% of the variance **(B)** Correlations using Spearmen tests between all dimensions (cytokine and lymphocyte dimensions) were reported.

Finally, we investigated whether there was a direct correlation between T lymphocyte improvements and the removal of inflammatory cytokines/chemokines consecutive to TPE treatment. For this purpose, we initially performed hypothesis-free PCAs with all the immunobiological data (cytokine plasma concentrations, immune cell frequencies and absolute numbers) collected from baseline to day 7 and subsequently estimated correlations between cytokine (from day 0 to day 4) and immune cell (from day 0 to day 7) variations. Three main dimensions were observed in respective PCAs, which discriminated three profiles of variations for both cytokine/chemokine and immune cell parameters (**Figure 9A**).

When considering cytokine/chemokine parameters, dimension 1 highlighted the weight of IL-1RA, IL-6 and TNF-α concentration variations, while dimensions 2 and 3 were mostly driven by CCL-2, IL-18, IL-10, CCL-3 and IL-7 or IFN-α, IFN-γ and IL-10 concentration variations, respectively. With regard to immune cell parameters, dimension 1 highlighted the increase in various T-cell populations, while dimension 2 corresponded mostly to CD4+ T cell changes and dimension 3 to changes in the frequencies of exhausted/senescent CD4+ and/or CD8+ T cell subsets.

An inverse correlation (r = -0.49) was recorded between the variations in CCL-2, IL-18, IL-10, CCL-3 and TNF-α concentrations (dimension 2, cytokine) and the increase in CD4+ T-cell subsets (dimension 2, immune cells) (**Figure 9B**). This result suggested that the removal of inflammatory cytokines by TPE may have helped the recovery of CD4+ T cells. In addition, a positive correlation was highlighted between the variations of cytokines from dimensions 2 & 3 and the decrease in frequencies of exhausted/senescent CD4+ and/or CD8+ T-cell subsets (r = 0.52 and r = 0.62, respectively). It should be noted that correlations were observed in the TPE group only, which suggests that excess cytokine/chemokine removal using TPE may have directly impacted the decrease in exhausted/senescent T cells observed in these patients.

## Discussion

Knowledge of immunity to severe COVID infections remains incomplete. The impact of therapies on initial immune perturbations merits deciphering in order to validate or invalidate their benefits and potential use. Our interventional study using TPE as an additional therapy performed early in severe COVID infections suggests the following: (1) TPE transiently eliminated anti-type I IFN auto-Abs and excess cytokines; (2) it quantitatively accelerated immune cell recovery; (3) it improved CD4+ T-cell numbers, CD4/CD8 T-cell ratio, naive T-cell numbers and restored conventional memory T-cell distribution; (4) it also triggered a decrease in the frequencies of activated, proliferating, exhausted and terminally-differentiated T-cell populations and (5) increased virus-specific CD4+ T-cell responses. Therefore, (6) despite no clear improvement in clinical parameters, TPE acted on the ongoing adaptive immune response, potentially restoring the conditions for an effective antiviral immune response.

Given the severity of COVID-19, indicators are essential for predicting disease outcomes. Of all the blood parameters (viral load, inflammatory or immune markers) assessed throughout COVID-19, circulating lymphocyte percentages and counts have proved to be the most sensitive parameters in predicting prognosis and patient outcome (12, 18–21). Hence, several studies demonstrated that the extent of lymphopenia at the onset of hospitalisation was clearly linked to poorer survival (12, 18–21). Conversely, early recovery of a normal blood lymphocyte count in the first week following hospitalisation was correlated with a reduction in disease severity and subsequent survival (40, 41). In this respect, we confirmed that patients receiving either TPE or ST had a better outcome (both on day 10 and after two months), when blood lymphocyte counts were significantly increased one week after ICU admission (**Figure 4**). Moreover, the addition of TPE to usual therapies led to subsequent increases in lymphocyte levels (**Figure 4**). This increase mainly concerned CD4+ T-cell count (**Supplementary Figure S5**) - a parameter that was reported to be strongly impaired in severe COVID infection (10, 13).

The importance of T-cell immunity for COVID-19 control has also been demonstrated in studies showing that patients with weak virus-specific T-cell responses failed to clear viral loads and consequently developed severe disease (5, 10, 42, 43). Delayed viral clearance has been associated with a paucity of naive T cells in particular (5, 10). The shortage of naive T cells has been emphasised in elderly patients who have significantly reduced naive T-cell repertoires, which explains why they were more prone to developing severe disease (10). In our study, naive CD8+ and, to a lesser extent, naive CD4+ T-cell counts were significantly higher on day 7 in patients with an early favourable outcome, and further increased with TPE (**Supplementary Figure S5**). These improvements may have helped reinvigorate the priming of T cells against SARS-CoV-2 (**Figure 8**) and/or co-morbidities.

In parallel, as demonstrated in various longitudinal studies characterising the T-cell immunophenotype during patient hospitalisation, an unusual distribution of memory T cells with a decrease in the number of Tcm and Tem cells and an increase in the percentage of terminally differentiated effector/memory cells (in both CD4+ and CD8+ T-cell subsets) has been recorded in many patients admitted to ICUs (11, 13, 44). Variations in T-cell differentiation patterns have also been correlated with dramatic increases in the frequency of activated and exhausted/senescent T-cell subsets. The latter are classically associated with defective T-cell responses and persist long-term in highly inflammatory environments, as described in COVID-19 patients. In this instance, we noted that TPE increased the number of CD4+ and CD8+ Tcm and Tem cells (**Supplementary Figures S5**, **7**) on day 7Moreover, TPE also induced a significant decline in exhausted/senescent CD4+ and CD8+ T cells (**Figures 6**, **7**) as corroborated by the few reports analysing the impact of therapeutic approaches on the immunity of hospitalised patients (45, 46). Hence, TPE may have helped to purge terminally differentiated cells impeding an effective anti-viral T-cell response (13, 47). In contrast, we observed that the frequencies of activated and proliferating cells (among CD4+ and CD8+ Tcm and Tem) remained relatively high on day 7 in both groups albeit with significant differences depending on treatment and early favourable or unfavourable outcome. An increase in an activated and proliferating cytotoxic CD8+ Tem subset was associated more specifically with the addition of TPE sessions, while elevated frequencies of Tcm and Tem cells lacking cytotoxic properties were observed more specifically in patients with an unfavourable early outcome (**Figures 6**, **7**). Whether activated, proliferating cytotoxic CD8+ Tem cells contained virus-specific CD8+ T cells or bystander CD8+ T cells that helped patients fight COVID-19 infection and, contrastingly, whether Tcm and Tem cells lacking cytotoxic properties corresponded to non-functional cells remains to be determined.

Having demonstrated accelerated recovery of immune cells during TPE, we then queried the key determinants capable of restoring effective immunity in these patients.

The purging of anti-type I IFN auto-Abs in COVID-19 patients is thought to restore effective type I IFN in these patients and enhance the antiviral T-cell response. In mouse models, type I IFN directly promotes T-cell survival and the differentiation of naive T-cell precursors into short-term CD8+ effector T cells active against virus-infected cells (48, 49). In our cohort, we observed the presence of anti-IFNω auto-Abs in fifty percent of the patients: 4 in the TPE group and 6 in the ST group. Interestingly, among the 4 TPE patients, the only one with a favourable outcome (TPE5) showed the greatest elimination of anti-IFNω auto-Abs, a robust improvement in T-cell recovery and a reduction in exhausted/senescent T cells. However, given the small patient cohort, no clear conclusions can be drawn regarding the potential impact of eliminating anti-IFNω auto-Abs on clinical benefit and restoration of an effective antiviral T-cell response.

The relationship between cytokine storm and lymphopenia has been studied extensively in cases of severe COVID infection (44, 50). Profound T-cell lymphopenia is thought to be a direct consequence of the release of inflammatory and homeostatic cytokines rather than sequestration of T cells in tissues and lymphoid organs (51). Hence, T-cell counts in COVID-19 were subsequently shown to be negatively correlated with IL-6, TNF-α and IL-10 blood levels, particularly in the elderly (44, 50). Hyperinflammation may cause lymphopenia by inducing apoptosis, pyroptosis (both due to prolonged secretion of TNF-α, IL-6 or IL-18), or PANoptosis (due to the synergistic effect of IFNγ and TNF-α) (17, 52, 53). In our study, we detected no variations in TNF-α, IL-6 or IFN-γ plasma concentrations but a dramatic drop in IL-18 and IL-7 between baseline and day 4 in the TPE-treated group compared to the ST-treated patients (**Figure 3**; **Supplementary Figure S4C**). These changes may have impacted the recovery of T-cell counts recorded after TPE. To this end, our correlation analyses between T lymphocyte improvements and the removal of inflammatory cytokines showed a strong correlation between variations in IL-18, IL-7 and, to a lesser extent, TNF-α and IFN-γ concentrations, and the increase in CD4+ T-cell counts (**Figure 9B**). It should be noted that IL-7 has long since been known to enhance the survival and maintain the size of naive T cells (54). In this case, we can assume that the initial increase in IL-7 was a consequence of its reduced consumption and excessive production by stromal cells, secondary to T-cell lymphopenia. By limiting inflammatory mediators such as IL-18, TPE may have helped restore a normal number of T cells, which consume IL-7 in order to proliferate.

In addition, beyond T-cell count recovery, excess cytokine removal may also have helped to reduce the frequency of dysfunctional T cells. Indeed, it has been suggested that IL-10 promotes T-cell exhaustion in COVID-19 (44). In this regard, variations in IL-10 concentrations (that decreased post-TPE) and, to a lesser extent, IFN-α and IFN-γ, were correlated with the decrease in exhausted T cells only after TPE.

Overall, our data showed that the removal of excess cytokines and/or anti-type I IFN auto-Abs may have improved several key determinants to restore effective immunity in TPE-treated patients.

Despite major improvements in T-cell metrics, TPE generated few clinical benefits in our small cohort of severely ill COVID-19 patients. Several factors may explain why the addition of TPE sessions did not alter the clinical course and severity of respiratory symptoms. The overall severity at baseline differed between the patient groups. Indeed, while lung function and related oxygenation parameters were found to be similar between TPE and ST patients, significant differences were recorded in terms of inflammatory and prothrombotic parameters (CRP and D-dimers, **Table 1**). This may have explained why TPE patients were more likely to develop complications. Indeed, a slightly higher rate of intubation and death was recorded at day 10 in the TPE group, although the addition of TPE resulted in the same survival rate at day 60 in both groups (**Figures 1E, F**). In addition, compared to ST, some TPE patients experienced severe early complications in the first few days of hospitalization (massive pulmonary embolism on day 1 for TPE2 which was not due to TPE sessions and to central catheter as the first TPE session was performed on peripheral veins, refractory infection which led to intubation on day 4 for TPE4). Thus, the fact that TPE2 and TPE4 were still alive on day 60 can be considered a satisfactory outcome, which was not highlighted by the criteria used in this study.

As mentioned above, several case reports, retrospective series and controlled or randomised trials have reported clinical improvement after TPE, taking into account oxygenation or survival parameters (28–38). Meta-analyses converge in considering that TPE reduced mortality in patients with moderate to critical COVID-19 (55, 56). Faqihi et al. treated 43 patients with TPE versus 44 patients receiving usual care and showed that TPE tended to reduce mortality at day 35 (21% vs 35%, p = 0.09) (29). However, another recent randomised trial, which included 11 patients in the control and TPE groups, did not record any clinical improvement (38), raising important questions about the conditions that may favour the effectiveness of TPE. Different factors are likely to influence the impact of TPE on clinical outcome: (i) the number of TPE sessions performed, (ii) the ARDS severity, iii) the nature of co-treatment and iv) the vaccination status of COVID-19 patients at the start of TPE sessions. Hence, it was observed that patients who received 5 sessions of TPE required less mechanical ventilation and were more likely to be extubated, compared with studies that performed 3 or fewer sessions of TPE (32, 35, 36), which showed insufficient clinical improvement (33, 34). In our study, it would have been interesting to perform additional sessions of TPE to maintain cytokine depletion and determine whether this accelerates immune recovery. Alternatively, in one study, TPE significantly improved oxygenation parameters and survival only in the subgroup of patients with severe ARDS (PaO2/FiO2<100) (33). In our work, we have shown clinical and immunological improvements in patients who required intermediate FiO2, developed moderate ARDS (82<PaO2/FiO2<166) and had a low neutrophil/lymphocyte ratio at day 0 (TPE 1, 5, 8, 10) (**Supplementary Figure 2**; **Figure 1**).

Our study was conducted from April 2021 to October 2022, a period during which only certain treatments were available. Dexamethasone was already widely used as part of the standard of care, unlike antiviral treatments which were used in countries other than France. Tocilizumab was also beginning to be used and vaccination against Covid-19 was gradually becoming available. In our cohort, four patients received tocilizumab: TPE7 (on day 3, several hours after the end of the last TPE session) and TPE10 as well as ST18 and ST20 (all on day 0). But, variable results and the small number of patients treated with tocilizumab prevented us to draw any definitive conclusions for a potential synergic effect with TPE. Of note, a randomised trial comparing tocilizumab, TPE or the TPE combination with tocilizumab did not show superiority in terms of clinical outcome or inflammatory parameters (34). Similarly, few patients (TPE6 and ST15 and ST19) in our cohort were vaccinated with mRNA Pfizer vaccine at least 15 days prior to ICU admission. Due to the low number of vaccinated patients, it was also not possible to draw any conclusions on the influence of vaccination on immune recovery.

Transfusion of convalescent plasma after completion of a single TPE session early after arrival in the ICU has been performed with encouraging results in terms of survival and oxygenation or inflammation parameters (57, 58). However, these studies did not include a TPE group comparator. It was therefore difficult to establish whether the combined effects of TPE plus convalescent plasma were superior to the use of TPE alone (57). Randomised trials comparing TPE plus convalescent plasma, TPE and standard care are urgently required to clarify this issue.

Finally, it would have been interesting to assess auto-Abs, cytokines and T-cell changes at different times after the TPE session (and not only on days 4 and 7), and to analyse complementary aspects of COVID immunity such as viral load and accumulation of resident memory T cells in the respiratory tract, etc. This would have probably helped us to better correlate the dynamics of auto-Abs/cytokine clearance, virus accumulation/elimination and improvement in cellular immunity with the evolution of clinical symptoms.

In conclusion, our results therefore show that the addition of TPE sessions to standard treatment accelerates immune cell recovery and contributes to the development of appropriate T-cell responses in patients suffering from severe COVID-19 disease, providing a sound basis for adding this therapy to anti-inflammatory treatments in the case of severe inflammatory infections.

## Data Availability

The raw data supporting the conclusions of this article will be made available by the authors, without undue reservation.
