# Supplementary material for: Therapeutic plasma exchange accelerates immune cell recovery in severe COVID-19
Source: Front Immunol. 2025 Jan 17;15:1492672. doi: 10.3389/fimmu.2024.1492672 (PMC11782122; doi:10.3389/fimmu.2024.1492672)

Supplementary Figure 1 : Study design. Treatment's schedule and sample collection for biological investigations.

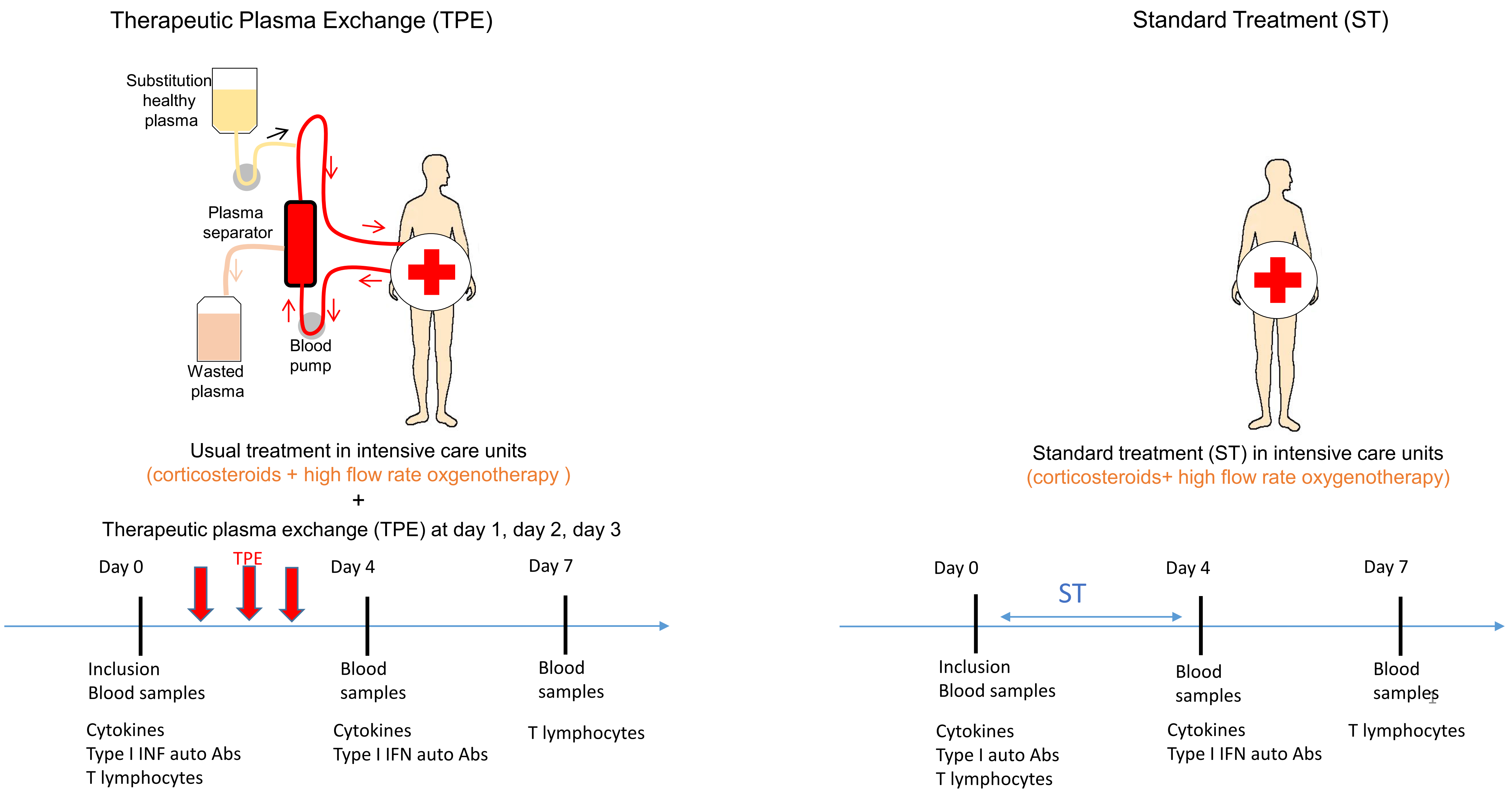

Supplement: Supplementary file 1 [file Image1.pdf]
