# Supplementary figures and images for: Therapeutic plasma exchange accelerates immune cell recovery in severe COVID-19
Source: Front Immunol. 2025 Jan 17;15:1492672. doi: 10.3389/fimmu.2024.1492672 (PMC11782122; doi:10.3389/fimmu.2024.1492672)

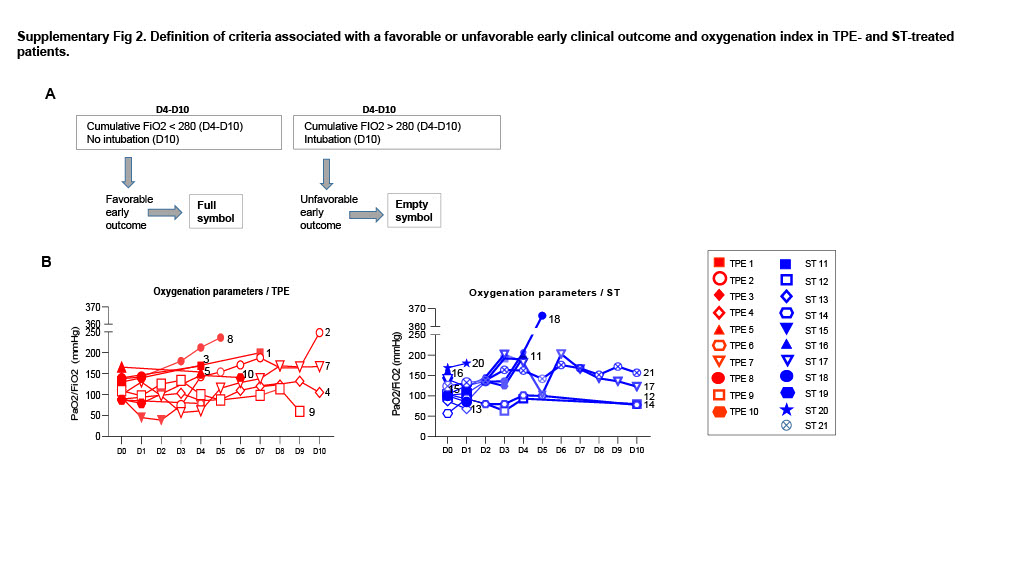

Supplement: Supplementary file 2 [file Image2.jpeg]

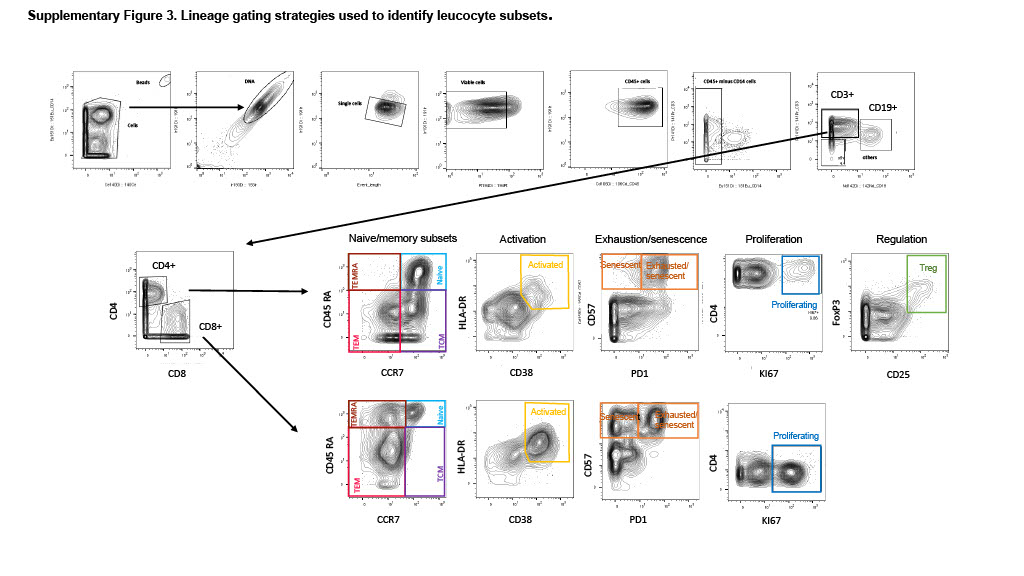

Supplement: Supplementary file 3 [file Image3.jpeg]

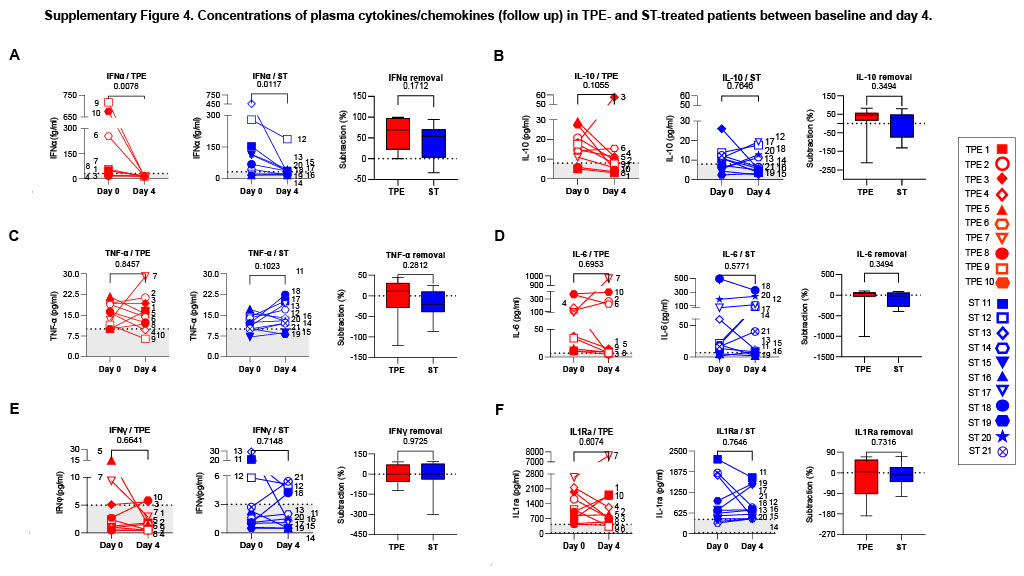

Supplement: Supplementary file 4 [file Image4.jpeg]

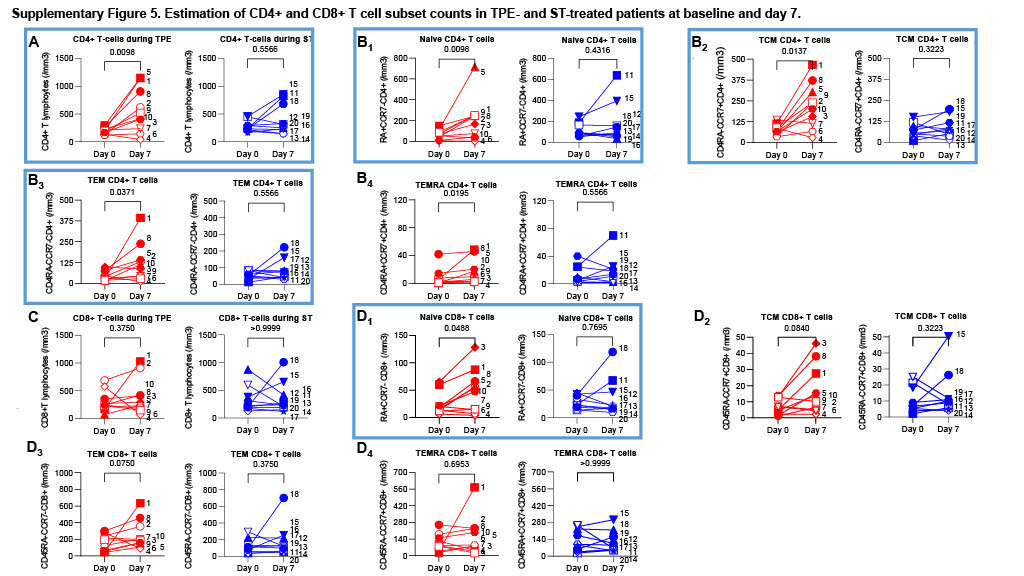

Supplement: Supplementary file 5 [file Image5.jpeg]

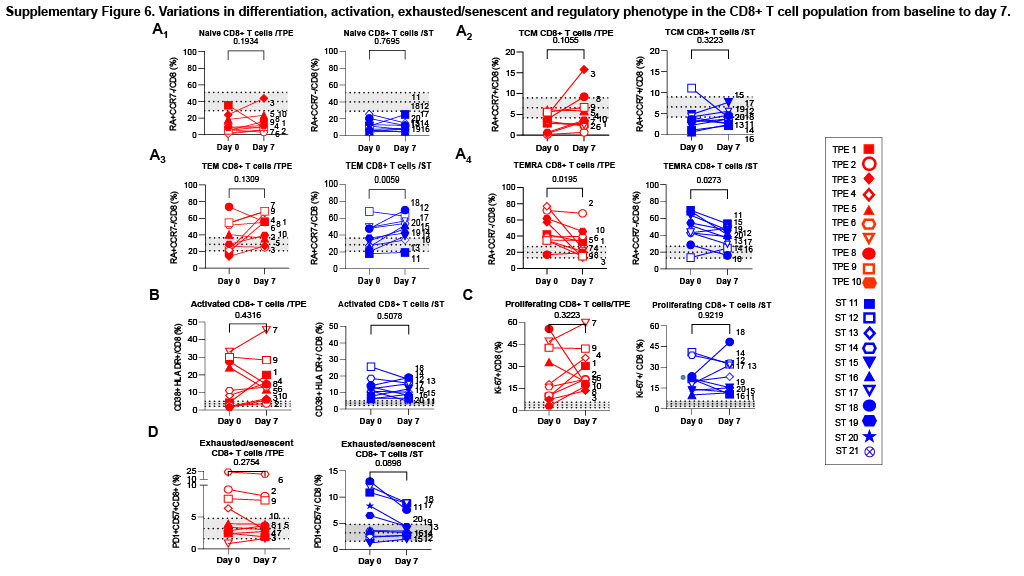

Supplement: Supplementary file 6 [file Image6.jpeg]
