# Supplementary material for: Therapeutic plasma exchange accelerates immune cell recovery in severe COVID-19
Source: Front Immunol. 2025 Jan 17;15:1492672. doi: 10.3389/fimmu.2024.1492672 (PMC11782122; doi:10.3389/fimmu.2024.1492672)
Supplement: Supplementary file 7 [file DataSheet1.pdf]

Supplementary Table 1: Patient's demographics, clinical features and treatments at baseline – Individual data

Patient	Gender	Age	Comorbidities	PaO2/FiO2 (%)	FiO2 (%)	Initial treatment
TPE 1	F	69	Overweight, asthma	130	50	dexamethasone
TPE 2	M	73	Diabetes	93	60	dexamethasone
TPE 3	M	57	HTA, nephrectomy, paraplegia	140	50	dexamethasone
TPE 4	M	64	HTA, overweight	89	93	methylprednisolone
TPE 5	M	65	HTA, overweight, diabetes	165	70	dexamethasone
TPE 6	M	87	HTA, diabetes, Parkinson	87	70	dexamethasone
TPE 7	M	70	HTA	100	60	dexamethasone, tocilizumab
TPE 8	M	61		138	50	dexamethasone
TPE 9	M	77	Arteriopathy	109	65	dexamethasone
TPE 10	M	38	Overweight, diabetes	88	60	dexamethasone, tocilizumab
ST 11	M	51	Overweight; HTA	101	70	dexamethasone
ST 12	M	55	Allergic rhinitis	104	70	dexamethasone
ST 13	F	56	Overweight	87	70	dexamethasone
ST 14	M	73		57	80	dexamethasone
ST 15	M	68	Overweight	132	50	dexamethasone
ST 16	M	64	Overweight, HTA, dyslipidemia	156	60	dexamethasone
ST 17	M	61		141	70	dexamethasone
ST 18	M	43		130	50	dexamethasone, tocilizumab
ST 19	F	75	HTA, overweight, diabetes, dyslipidemia	104	70	dexamethasone
ST 20	M	55		170	53	dexamethasone, tocilizumab
ST 21	M	76	Myasthenia	124	50	dexamethasone

F: Female

M: Male

HTA: Arterial hypertension
