# Supplementary material for: Therapeutic plasma exchange accelerates immune cell recovery in severe COVID-19
Source: Front Immunol. 2025 Jan 17;15:1492672. doi: 10.3389/fimmu.2024.1492672 (PMC11782122; doi:10.3389/fimmu.2024.1492672)
Supplement: Supplementary file 8 [file DataSheet2.pdf]

Table S2. List of reactives used.

Techniques	Products	Supplyer	Antibody	clone	Ref
Mass cytometry					
Barcoding	Anti-Human CD45 (HI30)-106Cd—100 Tests	Standard Biotools	CD45	HI30	3106001B
	Anti-Human CD45 (HI30)-110Cd—100 Tests	Standard Biotools	CD45	HI30	3110001B
	Anti-Human CD45 (HI30)-111Cd—100 Tests	Standard Biotools	CD45	HI30	3111001B
	Anti-Human CD45 (HI30)-112Cd—100 Tests	Standard Biotools	CD45	HI30	3112001B
	Anti-Human CD45 (HI30)-113Cd—100 Tests	Standard Biotools	CD45	HI30	3113001B
	Anti-Human CD45 (HI30)-114Cd—100 Tests	Standard Biotools	CD45	HI30	3114001B
	Anti-Human CD45 (HI30)-116Cd—100 Tests	Standard Biotools	CD45	HI30	3116001B
Labelling	Anti-Human CD3 (UCHT1)-141Pr—100 Tests	Standard Biotools	CD3	UCHT1	3141019B
	Anti-Human CD19 (HIB19)-142Nd—100 Tests	Standard Biotools	CD19	HIB19	3142001B
	Anti-Human HLA-DR (L243)-143Nd—100 Tests	Standard Biotools	HLA-DR	L243	3143013B
	Anti-Human CD69 (FN50)-144Nd—100 Tests	Standard Biotools	CD69	FN50	3144018B
	Anti-Human CD25 (2A3)-149Sm—100 Tests	Standard Biotools	CD25 (IL-2R)	2A3	3149010B
	Anti-Human CD14 (M5E2)-151Eu—100 Tests	Standard Biotools	CD14	M5E2	3151009B
	Anti-Human CD95/Fas (DX2)-152Sm—100 Tests	Standard Biotools	CD95/Fas	DX2	3152017B
	Anti-Human TIM-3 (F38-2E2)-153Eu—100 Tests	Standard Biotools	TIM-3	F38-2E2	3153008B
	Anti-Human CD279/PD1 (EH12.2H7)-155Gd—100 Tests	Standard Biotools	CD279 (PD-1)	EH12.2H7	3155009B
	Anti-Human CD197/CCR7 (G043H7)-159Tb—50 Tests	Standard Biotools	CD197 (CCR7)	G043H7	3159003A
	Anti-Human CD39 (A1)-160Gd—100 Tests	Standard Biotools	CD39	A1	3160004B
	Anti-Human KI-67 (B56)-161Dy—100 Tests	Standard Biotools	Ki-67	B56	3161007B
	Anti-Human FoxP3 (PCH101)-162Dy—50 Tests	Standard Biotools	FoxP3	PCH101	3162011A
	Anti-Human CD56 (NCAM16.2)-163Dy—100 Tests	Standard Biotools	CD56 (NCAM)	NCAM16.2	3163007B
	Anti-Human CD45RO (UCHL1)-164Dy—100 Tests	Standard Biotools	CD45RO	UCHL1	3164007B
	Anti-Human CD223/LAG3 (11C3C65)-165Ho—100 Tests	Standard Biotools	CD223/LAG-3	11C3C65	3165037B
	Anti-Human CD73 (AD2)-168Er—100 Tests	Standard Biotools	CD73 (Ecto-5-nucleotidase)	AD2	3168015B
	Anti-Human CD159a/NKG2a (Z199)-169Tm—100 Tests	Standard Biotools	CD159a (NKG2A)	Z199	3169013B
	Anti-Human CD45RA (HI100)-170Er—100 Tests	Standard Biotools	CD45RA	HI100	3170010B
	Anti-Human CD38 (HIT2)-172Yb—100 Tests	Standard Biotools	CD38	HIT2	3172007B
	Anti-Human Granzyme B (GB11)-173Yb—100 Tests	Standard Biotools	Granzyme B	GB11	3173006B
	Anti-Human CD4 (SK3)-174Yb—100 Tests	Standard Biotools	CD4	SK3	3174004B
	Anti-Human CD71 (OKT-9)-175Lu—100 Tests	Standard Biotools	CD71	OKT-9	3175011B

	Anti-Human CD127/IL-7Ra (A019D5)-176Yb—100 Tests	Standard Biotools	CD127 (IL-7Ra)	A019D5	3176004B
	Anti-Human CD16 (3G8)-209Bi—100 Tests	Standard Biotools	CD16	3G8	3209002B
	Cell-ID™ Cisplatin 194Pt—100 µL	Standard Biotools	/	/	201194
	Cell-ID Intercalator-Ir—500 µM	Standard Biotools	/	/	201192B
coupled antibodies					
145Nd	purified anti human CD8b antibody	eBioscience	CD8b	SIDI8BEE	14-5273-82
147Sm	purified anti human Granulysin antibody	R et D systems	Granulysin	polyclonal goat IgG	AF3138
148Nd	purified anti human CD88 (C5aR) antibody	Biolegend	CD88	S5/1 (Ms IgG2a)	344302
150Nd	anti-human CD28 pure, REA612, 0,1ml	Miltenyi Biotec	CD28	REA612	130-122-350
156Gd	anti human CD159c (NKG2C) pure,, REA205, 0,1ml	Miltenyi Biotec	NKG2c	REA205	130-122-278
158Gd	anti human CD57 pure, REA769, 0,1ml	Miltenyi Biotec	CD57	REA769	130-124-525
166Er	Anti-human KLRG1 pure, REA261, 0,1ml	Miltenyi Biotec	KLRG1	REA261	130-126-458
Coupling kit	Maxpar® X8 Antibody Labeling Kit, 145Nd—4 Rxn	Standard Biotools	145Nd	/	201145A
	Maxpar® X8 Antibody Labeling Kit, 147Sm—4 Rxn	Standard Biotools	147Sm	/	201147A
	Maxpar® X8 Antibody Labeling Kit, 148Nd—4 Rxn	Standard Biotools	148Nd	/	201148A
	Maxpar® X8 Antibody Labeling Kit, 150Nd—4 Rxn	Standard Biotools	150Nd	/	201150A
	Maxpar® X8 Antibody Labeling Kit, 156Gd—4 Rxn	Standard Biotools	156Gd	/	201156A
	Maxpar® X8 Antibody Labeling Kit, 158Gd—4 Rxn	Standard Biotools	158Gd	/	201158A
	Maxpar® X8 Antibody Labeling Kit, 166Er—4 Rxn	Standard Biotools	166Er	/	201166A
Colonnes	Amicon Ultra-0.5 Centrifugal Filter Unit 3 kDa, 0.5 mL	Merk Millipore	/	/	UFC500308
	Amicon Ultra-0.5 Centrifugal Filter Unit 50 kDa, 0.5 mL	Merk Millipore	/	/	UFC505008
	Tris(2-carboxyethyl)phosphine hydrochloride (TCEP), pH 7.0	ThermoFisher	/	/	646547 ou 77720
	Antibody Stabilizer Tris	Candor Biosciences	/	/	130 055
	diluant and concentrate of fixation / permeabilization	eBioscience	/	/	00-5521-00
peptide SARSCov2	PepTivator SARS-CoV-2 Prot_N, Research Grade, 6 nmol	Miltenyi Biotec	/	/	130-126-698
	PepTivator SARS-CoV-2 Prot_M, Research Grade, 6 nmol	Miltenyi Biotec	/	/	130-126-702
	PepTivator SARS-CoV-2 Prot_S Complete, research grade, 6nmol	Miltenyi Biotec	/	/	130-127-951
eStimulation	Monensin	BD Biosciences	/	/	554724
	Brefeldin	Sigma	/	/	B5936-200ul

	CD28 Monoclonal Antibody, functional grade	Thermofisher (Invitrogen)	CD28	CD28.2	16028985
	Ultra-LEAF purified anti-human CD49d Antibody	Biolegend	CD49d	9F10	304339
Labelling	FcR blocking Reagent, human	Mintenyi Biotec	/	/	130-059-901
	CD3 PE Texas red	Thermofisher (Invitrogen)	PE texas red	7D6	MHCD0317
	CD4 FITC	BD Biosciences	FITC	RPA-T4	555346
	CD8b BUV395	BD Biosciences	BUV395	RPA-T8	563795
	DUMP- CD14 APC Vio770	Mintenyi Biotec	APCVio770	REA599	130-110-522
	DUMP- CD56 APC Vio770	Mintenyi Biotec	APCVio770	REA196	130-114-548
	DUMP- CD19 APC Cy7	Biolegend	APC Cy7	HIB19	302218
	CD154 BV605	Biolegend	BV605	24-31	310826
	TNFα PercpCy5.5	BD Biosciences	percpCy5.5	MAb11	560679
	IL2 APC	Biolegend	APC	MQ1-17H12	500310
	DUMP- fixable viability dye eFluor 780	Thermofisher (Invitrogen)	APC Cy7	/	65-0865-14
Anti-type I IFN auto-Abs					
	IFN-α2	Miltenyi Biotec			
	IFN-β	Miltenyi Biotec			
	IFN-ω	Merck			
