# Supplementary material for: Therapeutic plasma exchange accelerates immune cell recovery in severe COVID-19
Source: Front Immunol. 2025 Jan 17;15:1492672. doi: 10.3389/fimmu.2024.1492672 (PMC11782122; doi:10.3389/fimmu.2024.1492672)
Supplement: Supplementary file 9 [file DataSheet3.pdf]

Supplementary Table 3. Early (day 10) and final (day 60) clinical outcome.

Patient	Symbol	Early outcome d10	Final outcome d60
TPE 1	■	Decrease in O2 required	O2 weaning
TPE 2	○	Intubation at d1 (*)	No O2 weaning but extubation
TPE 3	◆	Decrease in O2 required	O2 weaning
TPE 4	◇	Intubation at d4	No O2 weaning
TPE 5	▲	Decrease in O2 required	O2 weaning
TPE 6	⬡	Deceased at d10	Deceased at d10
TPE 7	▽	Intubation at d4	No intubation weaning
TPE 8	●	Decrease in O2 required	O2 weaning
TPE 9	□	Intubation at d9	Deceased at d16
TPE 10	⬢	O2 weaning	O2 weaning
ST 11	■	O2 weaning	O2 weaning
ST 12	□	High flow rate O2 required	Intubation at d15
ST 13	◇	High flow rate O2 required	O2 weaning
ST 14	⬡	High flow rate O2 required	Deceased at d16
ST 15	▼	O2 weaning	O2 weaning
ST 16	▲	O2 weaning	O2 weaning
ST 17	▽	Intubation at d1	No O2 weaning
ST 18	●	Decrease in O2 required	O2 weaning
ST 19	⬢	Decrease in O2 required	O2 weaning
ST 20	★	Decrease in O2 required	O2 weaning
ST 21	⊗	Intubation at d2	Deceased at d45

*TPE 2: Intubation was necessary after a very early massive pulmonary embolism while additional treatment using TPE was just started (when 1 TPE session was performed on peripheral veins).
